# Neurodevelopmental Disruption of Cortico-Striatal Function Caused by Degeneration of Habenula Neurons

**DOI:** 10.1371/journal.pone.0019450

**Published:** 2011-04-29

**Authors:** Young-A Lee, Yukiori Goto

**Affiliations:** Department of Psychiatry, McGill University, Montreal, Quebec, Canada; Louisiana State University Health Sciences Center, United States of America

## Abstract

**Background:**

The habenula plays an important role on cognitive and affective functions by regulating monoamines transmission such as the dopamine and serotonin, such that its dysfunction is thought to underlie a number of psychiatric conditions. Given that the monoamine systems are highly vulnerable to neurodevelopmental insults, damages in the habenula during early neurodevelopment may cause devastating effects on the wide-spread brain areas targeted by monoamine innervations.

**Methodology/Principal Findings:**

Using a battery of behavioral, anatomical, and biochemical assays, we examined the impacts of neonatal damage in the habenula on neurodevelopmental sequelae of the prefrontal cortex (PFC) and nucleus accumbens (NAcc) and associated behavioral deficits in rodents. Neonatal lesion of the medial and lateral habenula by ibotenic acid produced an assortment of behavioral manifestations consisting of hyper-locomotion, impulsivity, and attention deficit, with hyper-locomotion and impulsivity being observed only in the juvenile period, whereas attention deficit was sustained up until adulthood. Moreover, these behavioral alterations were also improved by amphetamine. Our study further revealed that impulsivity and attention deficit were associated with disruption of PFC volume and dopamine (DA) receptor expression, respectively. In contrast, hyper-locomotion was associated with decreased DA transporter expression in the NAcc. We also found that neonatal administration of nicotine into the habenula of neonatal brains produced selective lesion of the medial habenula. Behavioral deficits with neonatal nicotine administration were similar to those caused by ibotenic acid lesion of both medial and lateral habenula during the juvenile period, whereas they were different in adulthood.

**Conclusions/Significance:**

Because of similarity between behavioral and brain alterations caused by neonatal insults in the habenula and the symptoms and suggested neuropathology in attention deficit/hyperactivity disorder (ADHD), these results suggest that neurodevelopmental deficits in the habenula and the consequent cortico-striatal dysfunctions may be involved in the pathogenesis and pathophysiology of ADHD.

## Introduction

The habenula is a part of the dorsal diencephalic conduction system, comprising of two distinct nuclei, the medial (MHb) and lateral (LHb) habenula [1]. The MHb receives inputs mainly from the limbic system, and sends outputs to the interpeduncular nucleus [1], which in turn regulates activity of monoamine, such as dopamine (DA) and serotonin (5HT), neurons located in the ventral tegmetnal area/substantia nigra pars compacta and dorsal raphe, respectively [1]. The LHb receives inputs from the basal ganglia and limbic structures, and sends outputs directly or indirectly through the rostromedial tegmental nucleus to the midbrain nuclei where DA and 5HT neurons are located [1], [2], [3].

Because of the neural network organizations of the habenula as the intersection between the limbic system and monoamine neurons in the midbrain, the habenula plays a critical role on regulation of monoamine transmission in widespread brain areas including the prefrontal cortex (PFC) and nucleus accumbens (NAcc). Pharmacological inactivation of the LHb has been shown to increase DA release in the PFC and NAcc [4], [5], [6], suggesting that the LHb provides inhibitory tone on DA transmission. In contrast, studies have shown that lesion of the habenula decreases 5HT release in the striatum and hippocampus [7], whereas electrical stimulation of the LHb increases 5HT release in the striatum [8], suggesting that the LHb, and possibly the MHb, yields excitatory drive on 5HT release. In agreements of habenula regulation of multiple monoamine transmission in various brain areas, a number of studies have shown that habenula lesion in adult rodents causes an assortment of behavioral deficits such as attenuated reward seeking behavior [9], and disruptions of attention [10], behavioral inhibition [10], social behavior [11], sensory gating [11], emotional learning [12], and spatial long-term memory [11], [13]. However, the results are often inconsistent across studies (e.g. electrical stimulation of the LHb also attenuates reward seeking behavior [14]), which may be partly associated with the extent of lesion given in the habenula because of the small size of these nuclei (e.g. lesion only given in the LHb vs. lesion of both MHb and LHb).

Because of critical role of the habenula on monoamine transmission, it is no wonder that habenula deficits have been implicated in a number of psychiatric disorders. A recent functional imaging study has shown robust activation of the thalamic areas including the habenula when normal subjects make errors in the behavioral flexibility test; in contrast, such activation is absent in schizophrenia patients [15]. However, more recent data show evidence that structural abnormalities of the human habenular complex are present in affective disorders, but not in schizophrenia [16]. Decrease of the habenula volume has been also reported in bipolar patients [17]. Habenula deficits in affective disorders are further supported by the studies such as therapeutic effects of LHb stimulation in severe major depressive disorder [18] as well as alleviation of depressive behavior such as learned helpless with habenula lesion in animal models of depression [19], [20], whereas avoidance deficits resembling learned helpless is induced by habenula stimulation [20], [21]. Animal studies also suggest habenula deficit in drug addiction [22], [23], and potential site of its therapeutic treatments [24], [25], although there is also a controversial study showing lack of effect of habenula lesion on heroin-self administration in rats [26].

The monoamine systems are highly vulnerable to neurodevelopmental insults because of their continuing maturation process up until early adulthood [27]. Thus, it is known that brain damage given in the brain structures that are involved in regulation of monoamine transmission induced different outcomes depending on timing of lesion. For example, neonatal lesion given in the hippocampus, which regulates DA neuron activity [28] causes neurodevelopmental sequelae in widespread brain areas where DA innervates such as the PFC and NAcc, and consequent detrimental outcomes that are different from those produced by lesion given in adult brains [29]. Given that the habenula is an important brain region regulating monoamine transmission, neonatal damage in the habenula is expected to produce neurodevelopmentally-specific alterations that are different from those caused by adult habenula lesion.

In this study, we examined behavioral and brain alterations caused by neonatal insults in the habenula. In particular, we examined chemical lesion of the MHb and LHb in neonatal brains of rats. We expected that neonatal habenula lesion (NHL) caused behavioral alterations that were different from those induced by adult habenula lesion, and neurodevelopmentally-specific alterations (e.g. alterations presenting only at specific ages) in the brain areas such as the PFC and NAcc where monoamine neurons projects. In addition, there are a number of studies showing distinctions between the MHb and LHb [1], [30], [31]. MHb, but not LHb, neurons exhibit high nicotinic receptor expression throughout life from prenatal period to adulthood [32]. Given that the study by Carlson has shown that repeated systemic treatments of nicotine causes neurodegeneration selectively in the MHb [33], we also examined the effects of neonatal nicotine microinfusion into the habenula (NNH) of neonates. We hoped that NNH produced neurodegeneraiton selectively in the MHb, such that we were able to elucidate the impacts of neonatal insults in the MHb without affecting LHb.

## Results

### Neonatal habenula lesion

**Figure 1:**
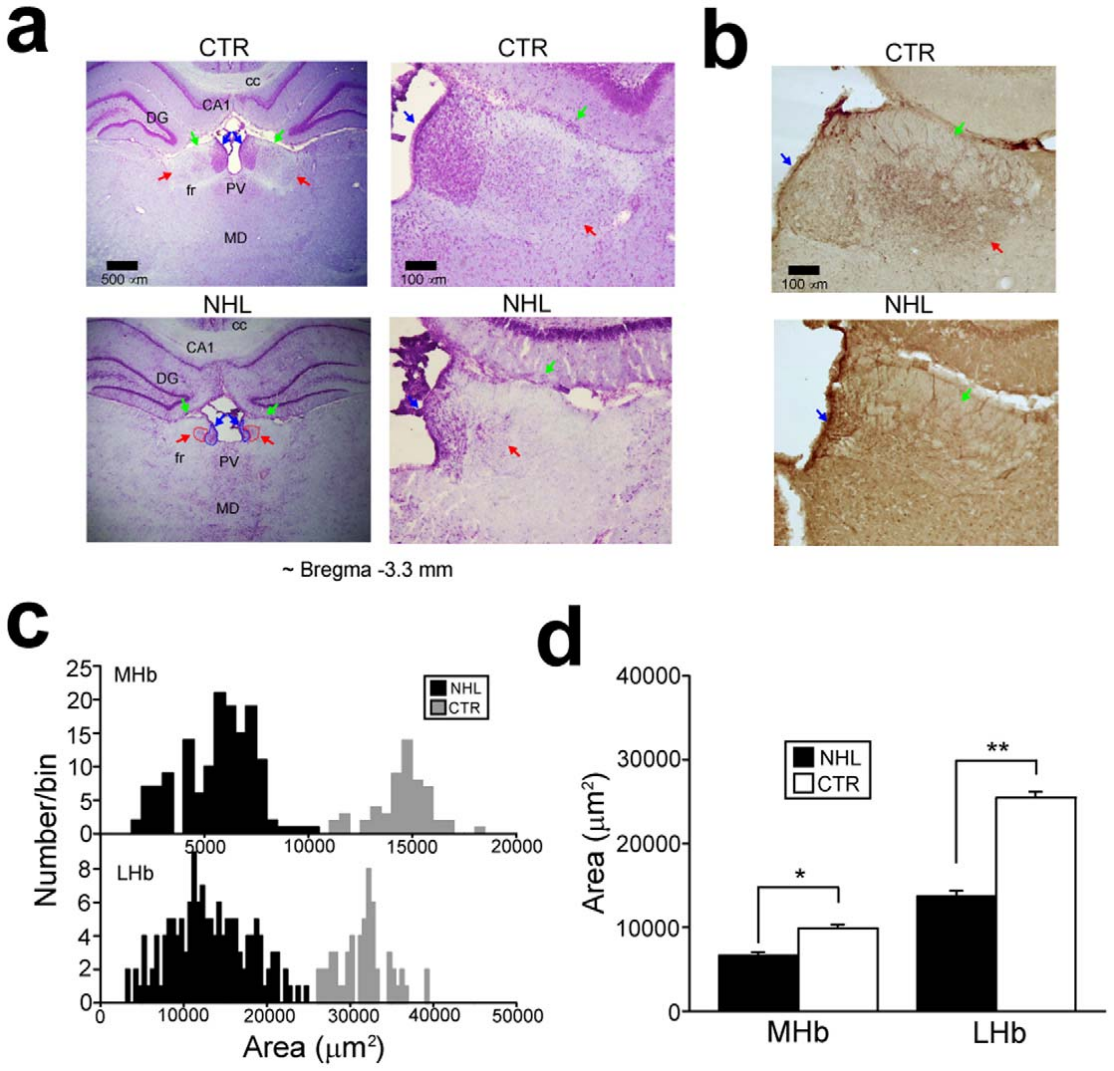
Histology of NHL. (**a**) Nissl stained sections of the habenula in an adult control (CTR; top) and NHL rats (bottom). The blue, red, and green arrows indicate the MHb, LHb, and stria medullaris. fr: fasciculus retroflexus; PV, paraventricular nucleus of the thalamus; MD, mediodorsal nucleus of the thalamus; LP, lateral posterior nucleus of the thalamus; DG, dentate gyrus; cc, corpus callosum. Scale bar = 500 and 100 µm for small and large magnification. (**b**) Sections with tyrosine hydroxylase immunostaining for the habenula nuclei. Tyrosine hydroxylase staining was observed in the LHb of CTR rats (top), whereas such staining was not present in NHL rats (bottom). This example also shows shrinkage of the MHb and LHb with NHL. (**c**) Histograms showing distribution of the areas of the MHb and LHb of adult NHL and CTR rats. (**d**) A Graph showing the areas of the MHb and LHb of adult NHL and CTR rats. *P<0.001, **P<0.001, with the post-hoc test.

NHL was conducted in male pups at postnatal day (PD) 7. NHL was examined when these rats reached adulthood (PD56 or older) with Nissl staining (Fig. 1a) and immunohistochemistry against tyrosine hydroxylase (Fig. 1b). NHL resulted in shrinkage of the MHb (NHL, 6623±127.6 µm^2^, n = 146; control sham lesion (CTR), 10402±180.3 µm^2^, n = 93; t_237_ = 16.1, P<0.001, unpaired t-test for NHL vs. CTR; Fig. 1c, d) and LHb (NHL, 13496±420.6 µm^2^, n = 146; CTR, 25213±434.4 µm^2^, n = 93; t_237_ = 16.0, P<0.001, unpaired t-test for NHL vs. CTR; Fig. 1c, d) nuclei. In all cases, both MHb and LHb were affected by NHL, and therefore, there was no animal in which only either the MHb or LHb was damaged. Larger lesions were accompanied with damages in other brain areas such as the dorsal hippocampus, stria medullaris, and thalamic nuclei. These animals were excluded from the data.

### Hyperlocomotion in juvenile NHL rats

**Figure 2:**
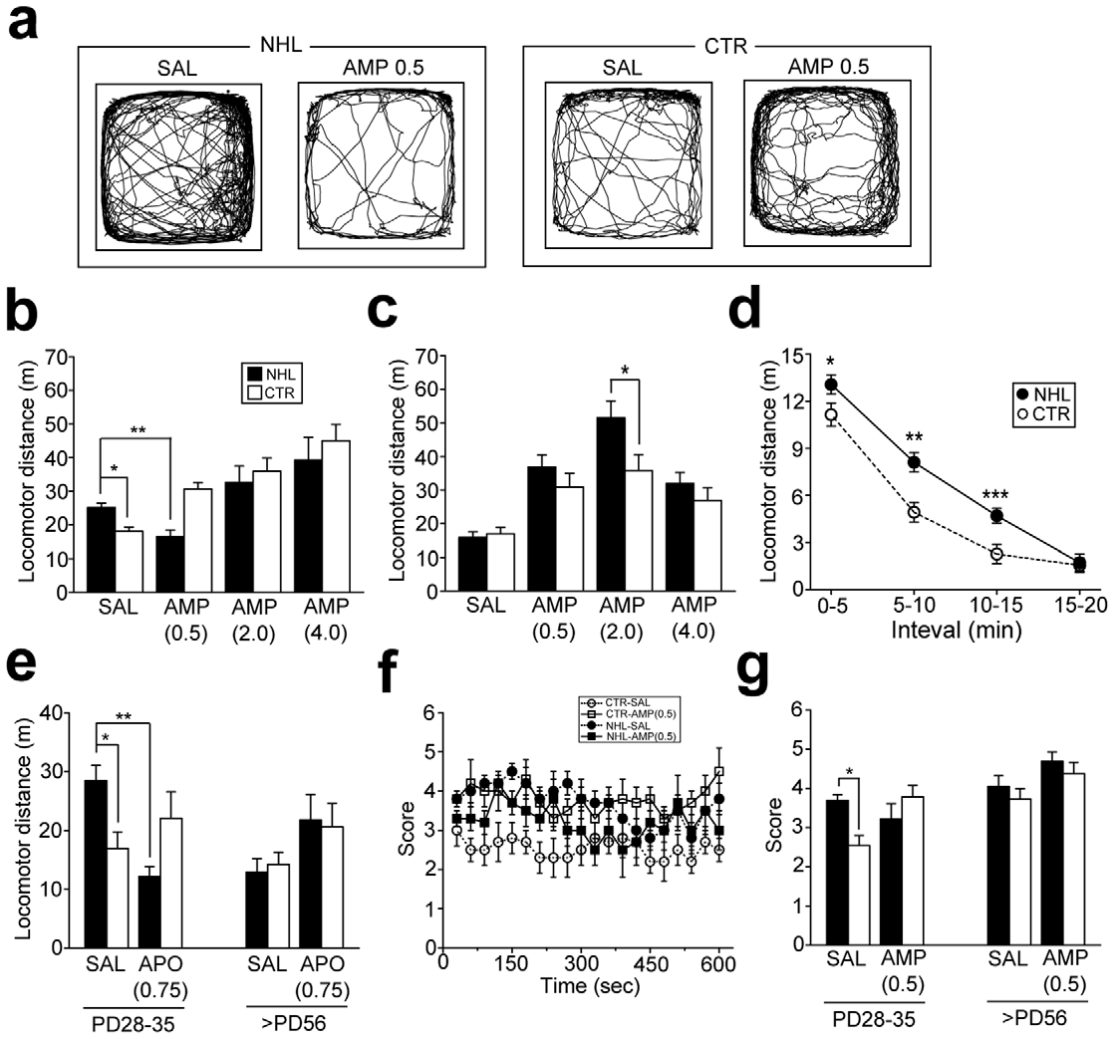
Hyperlocomotion and stereotypy in juvenile rats caused by NHL. (**a**) Representative examples of spontaneous locomotor movements of juvenile NHL rats and age-matched CTR receiving SAL or 0.5 mg/kg of AMP (AMP0.5). (**b**) A graph showing locomotor distance of juvenile NHL and CTR rats with SAL, or different doses of AMP (AMP0.5–4.0). *P = 0.041, **P = 0.038, with the post-hoc test. (**c**) A graph showing locomotor distance of adult NHL and CTR rats with SAL or AMP. *P = 0.002. (**d**) A graph showing 5 min segments of locomotor distances of juvenile NHL and CTR rats. *P = 0.024, **P<0.001, ***P = 0.005, vs. CTR. (**e**) APO (0.75 mg/kg; APO0.75) attenuated hyper-locomotion in juvenile NHL rats. *P = 0.010, **P<0.001. (**f**) A graph showing stereotypic behaviors scored every 30 seconds for 10 minutes in juvenile NHL and age-matched CTR rats receiving SAL and 0.5 mg/kg of AMP. (**g**) Stereotypy was increased in juvenile, but not adult, NHL rats compared to age-matched CTR rats. AMP did not attenuate stereotypy in NHL rats. *P = 0.003.

Behavioral impacts of NHL were first examined with the locomotion test when animals reached at PD28–35 (juvenile period) and PD56 or older (adulthood). Multivariate analysis of variance (ANOVA) revealed significant effects of interaction between lesion types (NHL vs. CTR) and ages of animals on locomotion (PD28 vs. PD56; F_1,197_ = 0.48, P = 0.490 for lesion; F_1,197_ = 0.07, P = 0.79 for ages; F_1,197_ = 9.56, P = 0.002 for lesion×ages). Post-hoc analysis revealed that total distance of locomotion in juvenile NHL rats (25.2±1.3 m, n = 25) was significantly higher than that in CTR rats (18.1±1.3 m, n = 22; P = 0.041 for NHL vs. CTR; Fig. 2a, b). However, such hyperlocomotion was not observed in adult NHL rats (NHL, 15.9±1.6 m, n = 15; CTR, 17.0±1.9 m, n = 12; Fig. 2c). Hyperlocomotion in juvenile NHL rats was novelty-dependent, as locomotor distance in these rats was higher than that in CTR rats only for the first 15 minutes after placement of animals into the chamber (Fig. 2d). To elucidate whether NHL-induced hyperlocomotion in juvenile rats was associated with altered DA transmission, we examined the effects of amphetamine (AMP) treatments. Statistical analysis revealed significant effects of AMP treatments on locomotion (F_3,197_ = 30.8, P<0.001 for AMP treatments; F_3,197_ = 1.63, P = 0.184 for AMP treatments×lesion types; F_3,197_ = 10.7, P = 0.002 for AMP treatments×ages; F_3,197_ = 4.51, P = 0.004 for AMP treatments×lesion types×ages). With post-hoc analysis, we found that a low dose (0.5 mg/kg, i.p.; 16.5±1.9 m, n = 12; P = 0.38 for saline (SAL) vs. AMP treatments; Fig. 2a, b), but not higher doses (2.0 and 4.0 mg/kg; Fig. 2b), of AMP attenuated hyperlocomotion in juvenile NHL rats. On the other hand, AMP treatments at any doses increased locomotion in adult NHL rats (Fig. 2c). Moreover, augmented AMP responses were observed in adult NHL rats with treatments of 2.0 mg/kg of the drug compared to CTR rats (NHL, 51.5±5.1 m, n = 12; CTR, 35.8±4.7 m, n = 11; P = 0.002 for NHL vs. CTR; Fig. 2c). To further elucidate whether NHL-induced locomotor alterations was DA-dependent, the effects of the non-selective DA receptor agonist, apomorphine (APO; 0.75 mg/kg), was examined. Similar to AMP, APO attenuated hyperlocomotion in juvenile NHL rats (12.2±1.7 m, n = 9; P<0.001 for SAL vs. APO treatments with post-hoc test; Fig. 2e).

We further analyzed stereotypy in NHL rats, as stereotypy has been also shown to be associated with the DA system [34]. ANOVA revealed significant effects of lesion and ages (F_1,20_ = 9.32, P = 0.006 for lesion types; F_1,197_ = 0.95, P = 0.006 for ages; F_1,197_ = 2.99, P = 0.099 for lesion types×ages). In particular, stereotypy was significantly more frequent in juvenile NHL rats (score developed by Creese and Iversen [34], 3.7±0.15, n = 6) than CTR rats (2.5±0.25, n = 6; P = 0.003 for NHL vs. CTR; Fig. 2f, g). However, different from hyperlocomotion, 0.5 mg/kg of AMP treatments did not attenuate increased stereotypy in juvenile NHL rats, whereas this dose of AMP significantly increased stereotypy in CTR rats (Fig. 2f, g). Stereotypy was not different between adult NHL rats (4.1±0.27, n = 6) and CTR rats (3.7±0.27, n = 6; Fig. 2g).

These results suggest that NHL induces DA-dependent locomotor changes in juvenile, but not adult, rodents.

### Impulsivity in juvenile NHL rats

Hyperlocomotion in juvenile, but not adult, NHL rats and the paradoxical effect of AMP on it were reminiscent of attention deficit/hyperactivity disorder (ADHD) symptoms and its pharmacotherapy [35]. Thus, we examined whether other behavioral changes associated with the ADHD symptoms, i.e. impulsivity and attention deficit, were also caused by NHL.

**Figure 3:**
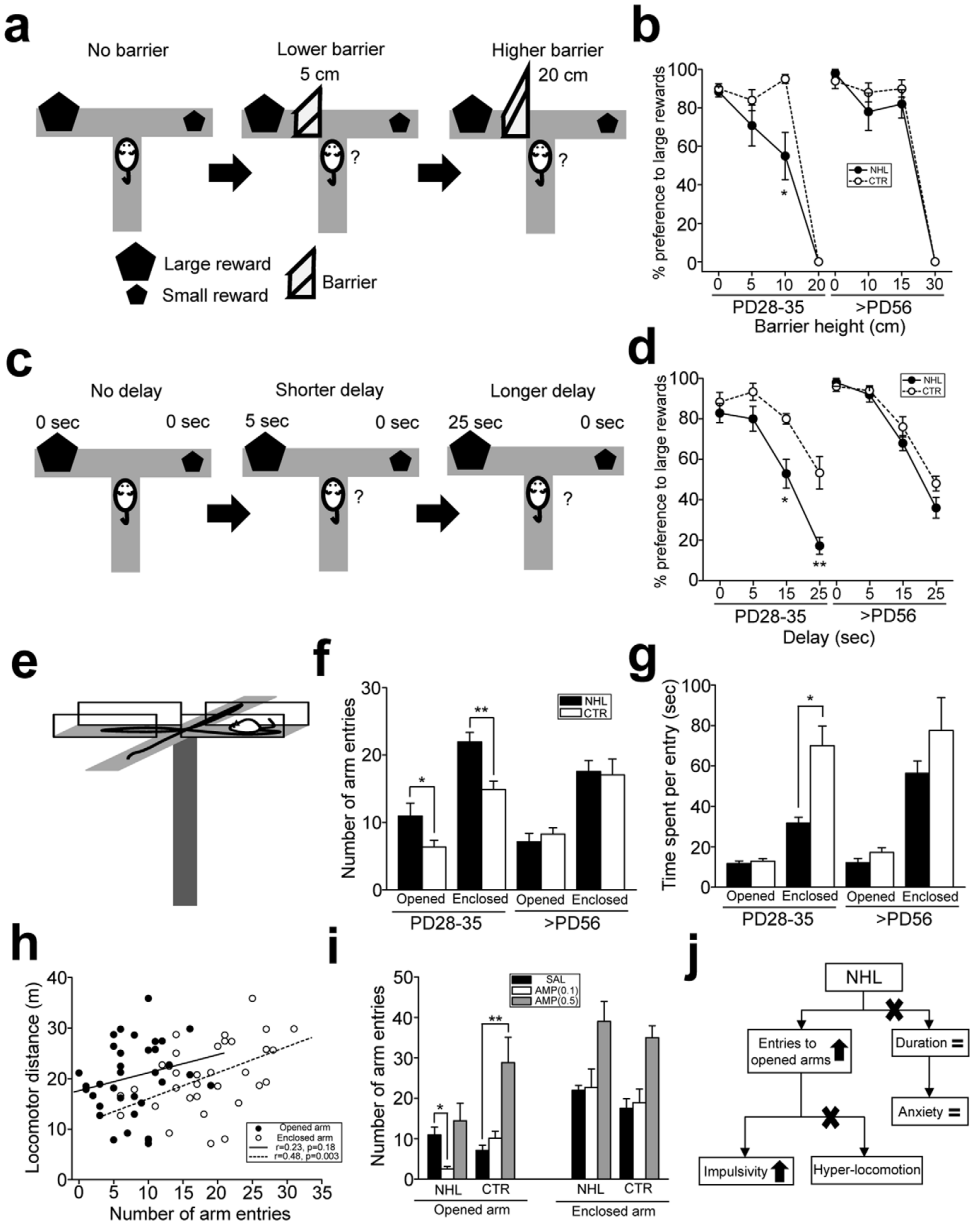
Impulsive behavior caused by NHL. (**a**) Schematic diagrams illustrating the effort-discounting test. (**b**) A graph showing preference to large rewards in the effort-discounting test with variable barrier heights. *P = 0.015, vs. CTR rats at 10 cm barrier. (**c**) Schematic diagrams illustrating the delay-discounting test. (**d**) A graph showing preference to large rewards in the delay-discounting test with variable delays. *P<0.001, **P<0.001, vs. CTR rats at 15 and 25 second delays, respectively. (**e**) A schematic diagram illustrating the EPM test. (**f**) A graph showing a number of entries into the opened and enclosed arms. *P = 0.028, **P = 0.001. (**g**) A graph showing time spent in the opened and enclosed arms per visit. *P<0.001. (**h**) A graph showing relationship between locomotion and a number of opened and enclosed arm entries in the EPM. (**i**) A graph showing modulation of the number of opened and enclosed arm entries by AMP in juvenile NHL and CTR rats. *P = 0.008, **P<0.001. (**j**) A schematic diagram illustrating the findings with the EPM test that indicates that increased opened arm entries are due to impulsivity.

Impulsivity was first assessed with the effort- (Fig. 3a) and delay- (Fig. 3c) discounting tests modified from those used in the study by Denk [36] using the T-maze. In the effort-discounting test, there was significant effect of lesion on performance of the test (F_1,84_ = 5.23, P = 0.025 for lesion types; F_1,84_ = 2.41, P = 0.123 for ages; F_1,84_ = 2.64, P = 0.077 for lesion types×ages). In particular, there was no difference in preference for large rewards between juvenile NHL and CTR rats in the sessions with no and 5 cm barrier (Fig. 3b). However, when 10 cm barrier was placed, juvenile NHL rats exhibited lower preference for large rewards (55.0±12.2% for large reward, n = 12) than CTR rats (95.0±2.2%, n = 10; P = 0.015 for NHL vs. CTR; Fig. 3b). In contrast, preference for large rewards in adult NHL rats was not different from that in CTR rats in any heights of the barrier (Fig. 3b). In the delay-discounting test, there were significant effects of lesion, ages, and their interaction on performance of the test (F_1,76_ = 25.4, P<0.001 for lesion types; F_1,76_ = 8.82, P = 0.004 for ages; F_1,76_ = 9.42, P = 0.003 for lesion types×ages). Post-hoc analysis revealed that there was no difference on preference for large rewards between juvenile NHL rats and CTR rats in the sessions with no and 5 second delay (Fig. 3d). However, when delays were prolonged to 15 and 25 seconds, juvenile NHL rats exhibited lower preference for large rewards than CTR rats (NHL, 52.9±7.1% for 15 sec delay, 17.1±4.2% for 25 sec delay, n = 7; CTR, 80.0±2.6% for 15 sec delay, 53.3±8.0% for 25 sec delay, n = 6; P<0.001 for NHL vs. CTR for each of 15 and 25 sec delay; Fig. 3d). None of delay conditions caused different preference for large rewards between adult NHL rats and CTR rats (Fig. 3d). These results suggest that juvenile, but not adult, NHL rats are more impulsive on reward seeking than CTR rats.

Since these impulsivity tests involve other cognitive processes such as learning, it was unclear whether behavioral alterations observed in NHL rats were due to impulsivity or reflection of learning deficit. Thus, we further examined impulsivity using the elevated plus maze (EPM) that did not involve learning (Fig. 3e, j, also see Materials & Methods, Suppl. Fig. S1). ANOVA revealed significant effects of lesion, ages, and their interaction on the number of opened arm entries in the EPM test (F_1,57_ = 6.49, P = 0.014 for lesion types; F_1,57_ = 9.55, P = 0.003 for ages; F_1,57_ = 9.16, P = 0.004 for lesion types×ages). In particular, the number of opened arm entries was increased in juvenile NHL rats (10.9±1.9, n = 18) compared to CTR rats (6.4±1.0, n = 17; P = 0.028 for NHL vs. CTR; Fig. 3f). On the other hand, time stayed in the opened arms at each entry was not different between NHL rats and CTR rats (Fig. 3g). These observations suggest that increased opened arm entry may not be due to decreased anxiety, since if animals were less anxious, they would spend more time in the opened arm (Fig. 3j, also see Suppl. Fig. S1). However, hyperlocomotion may also increase the number of arm entries (Fig. 3j). To test this possibility, we examined whether locomotor distance was correlated with opened and enclosed arm entries. Although a significant correlation was found between locomotor distance and enclosed arm entries, there was no correlation between locomotor distance and opened arm entries (Fig. 3h), suggesting that increased opened arm entries may not be due to hyperlocomotion, whereas increased enclosed arm entries may be associated with hyperlocomotion (Fig. 3j). Indeed, such hyperlocomotion was reflected as decrease of time spent in enclosed arms in juvenile NHL rats compared to CTR rats (Fig. 3g). There was no difference in the number of opened arm entries between adult NHL rats (7.1±1.2, n = 15) and CTR rats (8.3±0.9, n = 11; Fig. 3f). These results suggest that increased opened arm entries in juvenile NHL rats may be due to increased impulsivity (Fig. 3j). AMP treatments modulated increased opened arm entries in juvenile NHL rats (F_1,34_ = 12.2, P = 0.001 for AMP treatments; F_1,34_ = 0.56, P = 0.460 for AMP treatments×lesion types). However, the dose of AMP that was effective to attenuate hyperlocomotion (0.5 mg/kg) further increased opened arm entries (Fig. 3i). In contrast, even lower dose (0.1 mg/kg) of AMP decreased opened arm entries to the level of CTR rats (2.6±0.6, n = 9; P = 0.008 for SAL vs. AMP treatments; Fig. 3i).

Collectively, these results suggest that NHL induces increased impulsivity in juvenile, but not adult, animals. However, the mechanism underlying impulsivity appears to be different from that of hyperlocomotion, as the effective dose of AMP to improve impulsivity is different from that for hyperlocomotion.

### Attention deficit in juvenile and adult NHL rats

**Figure 4:**
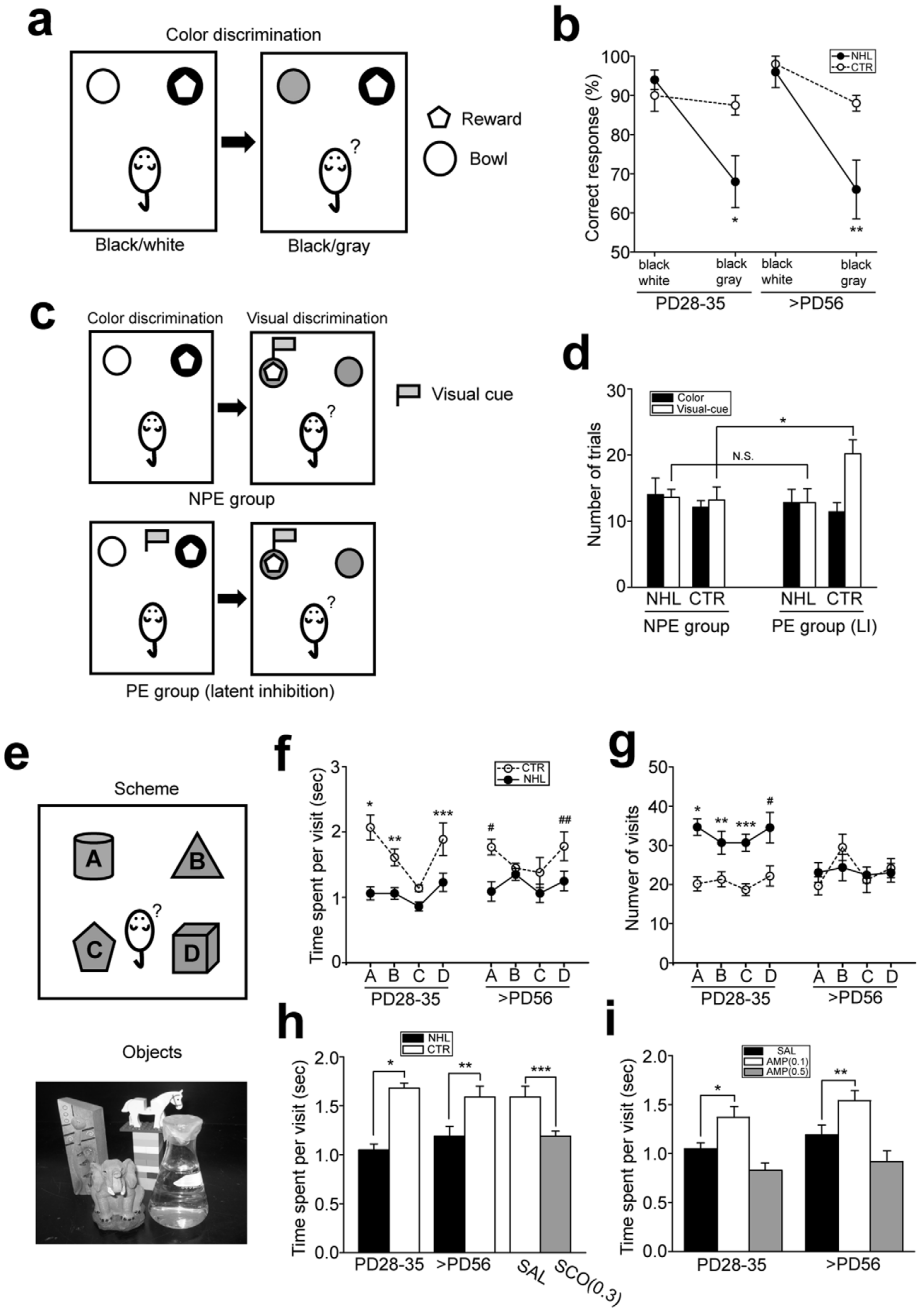
Attention deficit caused by NHL. (**a**) Schematic diagrams illustrating the color discrimination test. (**b**) A graph showing correct responses in the color discrimination test. *P = 0.006, **P = 0.001, vs. CTR rats in black-gray discrimination. (**c**) Schematic diagrams illustrating the experimental paradigm for color/visual discrimination tests for latent inhibition. NPE: non pre-exposed group to the conditioned stimulus, PE: pre-exposed group to the conditioned stimulus. (**d**) A graph showing a number of trials to reach the criterion in the LI test. *P = 0.029. (**e**) A schematic diagram illustrating object exploration test (top) and a photograph showing the objects used in the test (bottom). In this test, four distinct features of objects (A–D) were placed in each corner of the open field chamber. (**f**) A graph showing average time that NHL and CTR rats spent on exploration of the objects A–D per each visit. *P<0.001, **P = 0.009, ***P = 0.002, ^#^P = 0.001, ^##^P = 0.013 as opposed to CTR in each object. (**g**) A graph showing a number of visits that NHL and CTR made to the objects A–D. *P<0.001, **P = 0.011, ***P = 0.001, ^#^P = 0.001, as opposed to CTR in each object. (**h**) A graph showing time spent on exploration of objects. SCO: Scopolamine. *P<0.001, **P = 0.006, ***P = 0.048. (**i**) A graph showing AMP modulation of time spent on object exploration in NHL rats. *P = 0.014, **P = 0.008.

Attention deficit was first evaluated with the color discrimination test modified from that used in the study by Petrof and Brown [37] (Fig. 4a). Statistical analysis revealed significant effects of lesion on performance of the test (F_1,30_ = 9.58, P = 0.004 for lesion types; F_1,30_ = 0.44, P = 0.511 for ages; F_1,30_ = 0.44, P = 0.511 for lesion types×ages). In particular, in the black-white discrimination session, accuracy of correct bowl choice was similar between juvenile and adult NHL rats and age-matched CTR rats (Fig. 4b). However, in the gray-black session, in which discrimination required more attention than that for white-black, both juvenile and adult NHL rats (juvenile, 68.0±6.6%, n = 5; adult, 66.0±7.5%, n = 5) exhibited lower correct choice than age-matched CTR rats (juvenile, 87.5±2.5%, n = 4; adult, 88.0±2.0%, n = 5; P = 0.006 and P = 0.001 for juvenile and adult NHL vs. CTR, respectively; Fig. 4b).

We further examined attention deficit in adult NHL rats using the latent inhibition test [38] (Fig. 4c, also see Materials & Methods). In animals that were not exposed to the interference cue (NPE group) during color discrimination test, the number of trials to reach the criterion for the visual cue discrimination test was not different between NHL rats and CTR rats (Fig. 4d). In contrast, CTR rats that were pre-exposed to the interference cue (PE group; 20.1±2.1 trials, n = 5) took a significantly larger number of trials to reach the criterion in the visual cue discrimination test compared to those in NPE group (12.8±2.1 trials, n = 5; t_8_ = 2.48, P = 0.038, unpaired t-test for PE vs. NPE group; Fig. 4d), indicating the effects of latent inhibition. However, the number of trials to reach the criterion in the visual discrimination test in NHL rats of the PE group (13.6±1.2 trials, n = 5) was not different from those in the NPE group, indicating latent inhibition deficit (13.2±2.0 trials, n = 5; t_8_ = 0.17, P = 0.87, unpaired t-test for PE vs. NPE group; Fig. 4d).

Similar to the effort- and delay-discounting tests for impulsivity, these attention tests involved cognitive processes such as learning. Thus, we further examined attention deficit with the behavioral test that did involve learning process. The ability of sustained attention was further examined with the object exploration test modified from that used in the study by Lukaszewska [39] (Fig. 4e, also see Materials & Methods). ANOVA revealed significant effects of lesion on performance of this test (F_1,26_ = 24.5, P<0.001 for lesion types; F_1,26_ = 0.06; P = 0.814 for ages; F_1,26_ = 1.14, P = 0.296 for lesion types×ages). Post-hoc analysis revealed that juvenile NHL rats (1.05±0.06 sec, n = 6) spent significantly shorter time on object exploration compared to CTR rats (1.68±0.05 sec, n = 6; P<0.001 for NHL vs. CTR; Fig. 4f, h). A reduction of time for object exploration was also observed in adult NHL rats (1.19±0.10 sec, n = 12) compared to CTR rats (1.59±0.11 sec, n = 6; P = 0.006 for NHL vs. CTR; Fig. 4f, h). Duration of time spent on object exploration may be also influenced by motivation of animals to explore them. To address this issue, the number of visits to objects was also counted. The number of visits to each object was significantly increased in juvenile NHL rats compared to CTR rats, whereas the number of visits was not different between adult NHL rats and CTR rats (Fig. 4g). These results suggest that reduction of time for object exploration was not due to decreased motivation to explore the objects. AMP treatments also modulated object exploration in dose-dependent manner (F_1,37_ = 14.1, P<0.001 for AMP treatments; F_1,37_ = 0.05, P = 0.953 for AMP treatments×ages), with 0.5 mg/kg of AMP treatments further decreasing duration of object exploration (juvenile, 0.83±0.07 sec, n = 7; adult, 0.92±0.11 sec, n = 6; Fig. 4i), whereas 0.1 mg/kg of AMP treatments increasing duration of exploration to the level of CTR rats (juvenile, 1.37±0.11 sec, n = 6; adult, 1.54±0.10 sec, n = 6; P = 0.014 and P = 0.008 for SAL vs. AMP treatments in juvenile and adult NHL rats, respectively; Fig. 4i).

Collectively, these results suggest that NHL induces attention deficits, which are present both in juvenile period and adulthood.

### No effect of NHL on long-term memory

**Figure 5:**
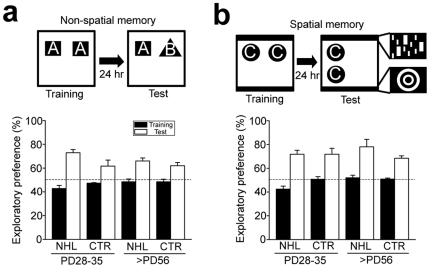
No effects of NHL on long-term memory. (**a**) A schematic diagram of non-spatial memory test (i.e. object recognition test; top) and a graph showing exploratory preference of previously exposed and novel objects (bottom). (**b**) A schematic diagram of spatial memory (novel place recognition test; top) test and a graph showing exploratory preference of objects located in the same and different places (bottom).

Non-spatial and spatial long-term memories were examined with the object recognition test and its modified version, novel place recognition test, respectively [40] (Fig. 5a, b). There was no difference in performance of these memory tests between NHL rats and CTR rats (Fig. 5a, b).

### Altered DA receptor and transporter expressions in the PFC and NAcc

**Figure 6:**
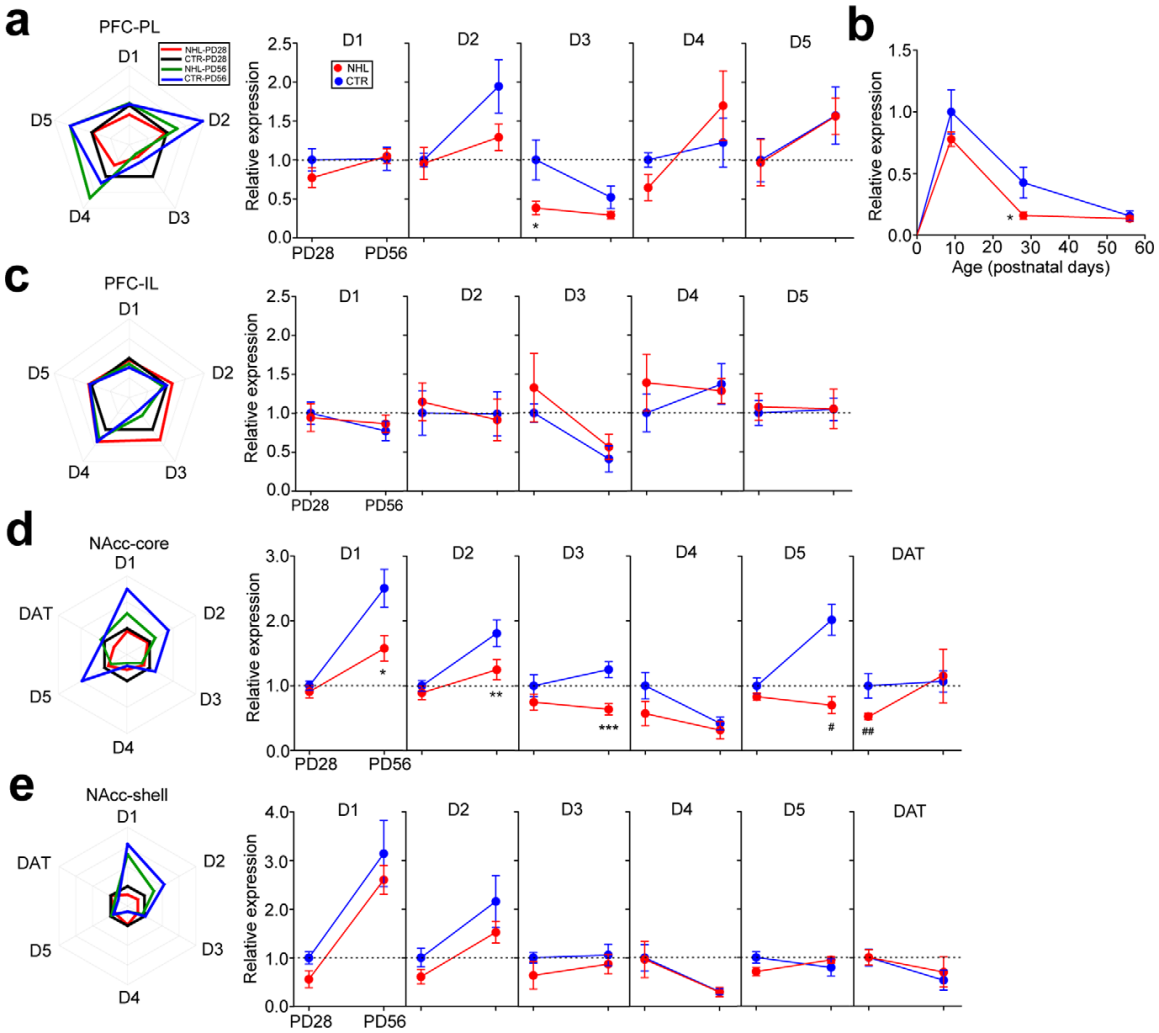
NHL-induced alterations of DA receptor and DAT expressions in the PFC and NAcc. (**a**) Graphs showing DA D1–D5 receptor expressions in the PL of NHL and CTR rats. All expressions were represented as relative to that in juvenile CTR rats. *P = 0.006. (**b**) A graph showing neurodevelopmental changes of DA D3 receptor expression and the effects of NHL on it. *P = 0.006. (**c**) Graphs showing no difference of DA D1–D5 receptor expression in the IL of NHL and CTR rats. (**d**) Graphs showing DA D1–D5 receptor and DAT expressions in the NAcc core of NHL and CTR rats. *P = 0.001, **P = 0.012, *** P = 0.001, ^#^P<0.001, ^##^P = 0.044, vs. CTR. (**e**) Graphs showing no difference of DA D1–D5 receptor and DAT expressions in the NAcc shell of NHL CTR rats.

To understand the mechanisms that underlay NHL-induced behavioral alterations and whether brain alterations caused by NHL were also consistent with those implicated in ADHD, we first examined whether NHL affected DA receptor and transporter (DAT) expressions in the PFC and NAcc. Using quantitative PCR for assays of DA receptor mRNA expression in the PFC, it was found that D3 receptor expression was significantly decreased in the prelimbic cortex (PL) of juvenile, but not adult, NHL rats (expression relative to juvenile CTR rats, juvenile, 0.38±0.09, n = 8; adult, 0.29±0.05, n = 11) compared to CTR rats (juvenile, 1.00±0.26, n = 7; adult, 0.52±0.14, n = 9; F_1,31_ = 4.35, P = 0.045 for lesion types; F_1,31_ = 9.52, P = 0.004 for ages; F_1,31_ = 2.00, P = 0.167 for lesion types×ages; P = 0.006 for juvenile NHL vs. CTR rats with post-hoc test; Fig. 6a, b). Further investigation revealed that D3 receptor expression in the PFC was the highest during the early postnatal period, and declined significantly through brain maturation into adulthood, which resulted in disappearance of different D3 receptor expression between NHL and CTR rats at adult (Fig. 6b). Furthermore, tendency of decreased D3 receptor expression was already apparent two days after lesion in NHL rats (i.e. PD9; Fig. 6b). On the other hand, no DA receptor expression was altered in the infralimbic cortex (IL; Fig. 6c).

In the NAcc core, DAT expression was selectively decreased in juvenile, but not adult, NHL rats (juvenile, 0.56±0.04, n = 18; adult, 1.15±0.41, n = 6) compared to CTR rats (juvenile, 1.00±0.16, n = 8; adult, 1.07±0.16, n = 10; F_1,45_ = 8.71, P = 0.005 for lesion types; F_1,45_ = 1.74, P = 0.194 for ages; F_1,45_ = 0.22, P = 0.641 for lesion types×ages; P = 0.044 for juvenile NHL vs. CTR rats with post-hoc test; Fig. 6d). In addition, D1, D2, D3, and D5 receptor expressions were also decreased in the NAcc core; however, these decreases were present only in adult NHL rats (Fig. 6d). No DA receptor or DAT expression was altered in the NAcc shell of NHL rats (Fig. 6e).

Since discrepancy between mRNA and protein expressions is frequently observed problem [41], we also examined protein expressions of D3 receptor in the PL and DAT in the NAcc core of juvenile NHL and CTR rats using western blot technique. PL D3 receptor (NHL, 0.52±0.010, n = 6; CTR 0.62±0.044, n = 6; t_10_ = 2.28, P = 0.046 for NHL vs. CTR; Suppl. Fig. S2a) and NAcc core DAT expressions (NHL, 0.17±0.008, n = 5; CTR, 0.20±0.013, n = 5; t_8_ = 2.73, P = 0.026 for NHL vs. CTR; Suppl. Fig. S2b) were significantly decreased in juvenile NHL rats compared to CTR rats.

These results suggest that decreased PL D3 receptor and NAcc core DAT expressions may be associated with hyperlocomotion and impulsivity induced by NHL, because of presence of these alterations only during the juvenile period.

### Reduction of PFC volume with NHL

**Figure 7:**
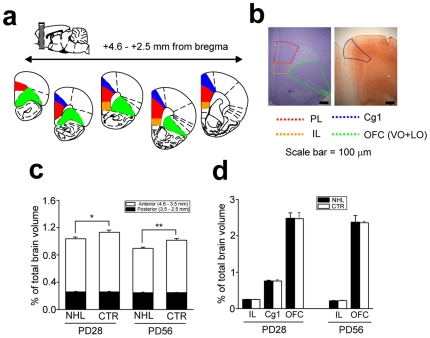
PFC volume reduction caused by NHL. (**a, b**) Schematic diagrams (a) and photographs (b) showing the brain areas measured. The volumes of the PL, IL, and OFC, were measured in the histological sections with Nissl staining, whereas the volume of Cg1 was measured in the sections with immunohistochemistry against SMI-32. (**c**) A graph showing the volume of the PL. White and black bars indicate anterior and posterior parts of the PL, respectively. *P = 0.020, **P = 0.017. (**d**) A graph showing no change in the volumes of IL, OFC, and Cg1 by NHL.

Accumulating evidence suggests that ADHD neuropathology involves decreased cortical thickness including the PFC [42], [43], [44], [45], [46]. Thus, we examined whether the volume of the PFC in NHL rats was decreased (Fig. 7a, b). A significant reduction of the PL volume was observed in both juvenile (percentage of PFC volume relative to a whole brain, 1.03±0.03%, n = 6) and adult (0.90±0.02%, n = 5) NHL rats compared to CTR rats (juvenile, 1.14±0.04%, n = 5; adult, 1.02±0.03%, n = 4; F_1,16_ = 13.8, P = 0.002; F_1,16_ = 18.5, P<0.001; F_1,16_ = 0.06, P = 0.810; P = 0.020 and P = 0.017 for juvenile and adult NHL vs. CTR rats, respectively, with post-hoc test; Fig. 7c). Further analysis revealed that the PL volume reduction was more prominent in the anterior than posterior parts (Fig. 7c). NHL did not alter the volumes of other cortical regions including the IL, anterior cingulated (Cg1), and orbitofrontal cortex (OFC; Fig. 7d).

These results suggest that NHL induces volumetric reduction selectively in the PL of NHL rats, which was present both in the juvenile period and adulthood.

### Correlations between behavioral alterations and alterations in the PFC and NAcc

**Figure 8:**
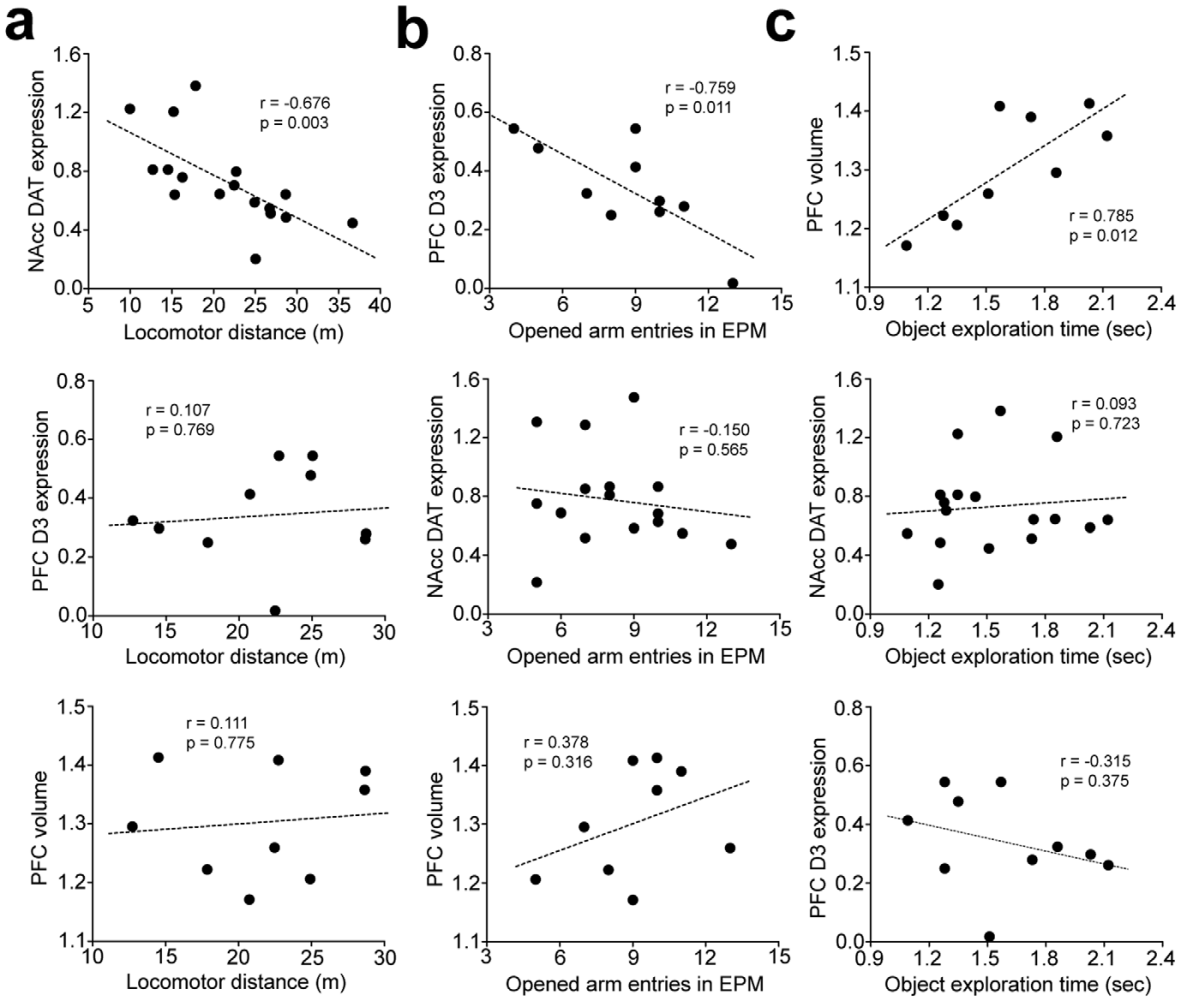
Correlation analyses of behavioral and brain alterations caused by NHL. (**a**) Graphs showing linear correlation between locomotor distance and DAT expression in the NAcc (top), but not D3 expression in the PFC (middle) or PFC volume (bottom). (**b**) Graphs showing linear correlation between opened arm entries in the EPM and D3 expression in the PFC (top), but not DAT expression in the NAcc (middle) or PFC volume (bottom). (**c**) Graphs showing linear correlation between exploration time in the object exploration test and PFC volume (top), but not DAT expression in the NAcc (middle) or D3 expression in the PFC (bottom).

To examine whether volume reduction and decreased D3 receptor expression in the PFC and decreased DAT expression in the NAcc are associated with hyperlocomotion, impulsivity, and attention deficit, we conducted correlation analyses between behavioral and brain alterations. Significant correlation was observed between DAT expression in the NAcc and hyperlocomotion (i.e. total locomotor distance) in juvenile NHL rats (r = −0.68, P = 0.003; Fig. 8a). On the other hand, correlations were observed between D3 expression in the PFC (r = −0.76, P = 0.011; Fig. 8b) and impulsivity as well as between PFC volume and attention deficit (r = 0.79, P = 0.012; Fig. 8c).

Taken together with developmentally-specific patterns of NHL-induced alterations (presence in only juvenile period vs. both in juvenile period and adulthood), these results suggest that NHL-induced behavioral alterations are caused by the PFC and NAcc alterations that are secondary to NHL.

### Neonatal nicotine administration into the habenula

**Figure 9:**
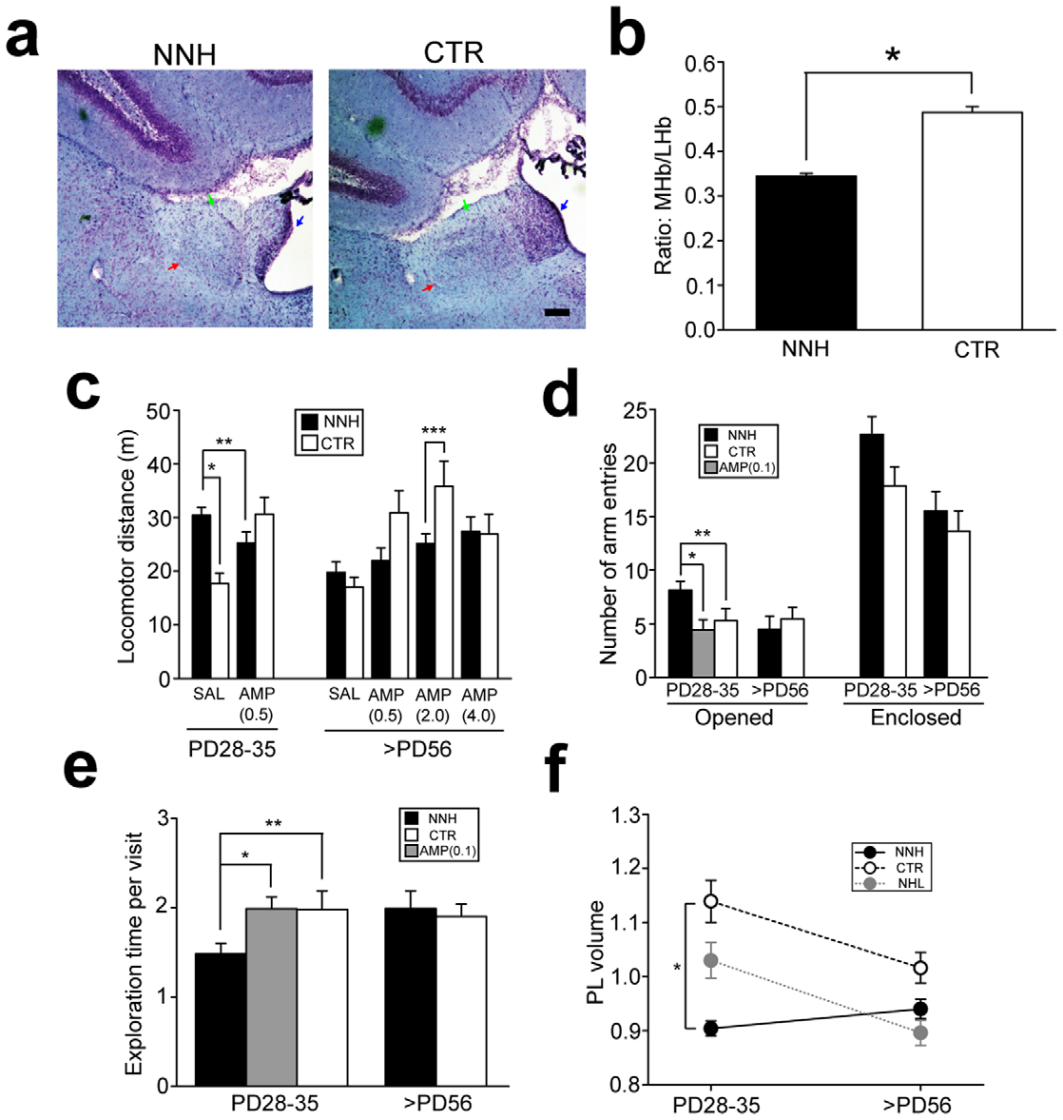
NNH-induced alterations. (**a**) An example of shrinkage of the MHb with NNH. This example shows one of the largest MHb shrinkage, which accompanied with consequent structural change in the LHb. The blue, red, and green arrows indicate the MHb, LHb, and stria medullaris, respectively. Scale bar = 100 µm. (**b**) A Graph showing the ratio of the areas of the MHb over LHb in adult NNH and CTR rats. *P<0.001. (**c**) A graph showing locomotor distance of juvenile and adult NNH and CTR rats with SAL, or different doses of AMP (AMP0.5–4.0). *P<0.001, **P = 0.008, ***P = 0.002. (**d**) A graph showing a number of opened and enclosed arm entries in the EPM. **P = 0.019, **P = 0.023. (**e**) A graph showing time spent on exploration of objects. *P = 0.014, **P = 0.013. (**f**) A graph showing the volume of the PL. *P<0.001. The PL volume of NHL rats shown in Fig. 7c is included in this graph to illustrate difference between NNL and NHL treatments on PL volume reduction with development.

One major caveat of our finding is that NHL affected both the MHb and LHb, and therefore it is unclear whether alterations we observed required neurodegeneration in both nuclei. To address this issue, we examined behavioral effects of NNH. NNH resulted in smaller size of the MHb (7742±188.9 µm^2^, n = 81; t_172_ = 14.7, P<0.001 for NNH vs. CTR; Fig. 9a), but not LHb (24464±495.9 µm^2^, n = 81; Fig. 9a), nuclei than those in CTR rats, which resulted in significant reduction of the ratio of the MHb over the LHb (ratio of MHb/LHb for NNH, 0.34±0.01; CTR, 0.49±0.01; t_116_ = −11.4, P<0.001 for NNH vs. CTR; Fig. 9b). Neuronal densities of the LHb nuclei in NNH and CTR rats were not different between NNH and CTR rats (NNH, 3.08±0.24, n = 81; CTR, 3.14±0.16, n = 93; Suppl. Fig. S3), excluding the possibility that NNH resulted in smaller neuronal size and greater packing of neurons.

NNH produced hyperlocomotion (NNH, 30.4±1.5 m, n = 16; F_1,50_ = 18.2, P<0.001 for lesion types; F_1,50_ = 9.81, P = 0.003 for ages; F_1,50_ = 7.65, P = 0.008 for lesion types×ages; P<0.001 for NNH vs. CTR rats with post-hoc test; Fig. 9c) and behavioral alterations indicating impulsivity (NNH, 8.1±0.8, n = 15; F_1,43_ = 9.59, P = 0.003 for lesion types; F_1,43_ = 4.91, P = 0.032 for ages; F_1,43_ = 8.61, P = 0.005 for lesion types×ages; P = 0.019 for NNH vs. CTR rats with post-hoc test; Fig. 9d) and attention deficit (NNH, 1.48±0.12 sec, n = 7; F_1,25_ = 8.22, P = 0.010 for lesion types; F_1,25_ = 1.71, P = 0.206 for ages; F_1,25_ = 21.0, P<0.001 for lesion types×ages; P = 0.014 for NNH vs. CTR rats with post-hoc test; Fig. 9e) in the EPM and object exploration tests, respectively, in juvenile rats. These NNH-induced hyperlocomotion (25.2±2.1 m with 0.5 mg/kg of AMP, n = 16; F_1,50_ = 17.3, P<0.001 for AMP treatments; F_1,50_ = 26.9, P<0.001 for AMP treatments×lesion types; F_1,50_ = 2.11, P = 0.153 for AMP treatments×ages; F_1,50_ = 1.26, P = 0.267 for AMP treatments×lesion types×ages; P = 0.008 for SAL vs. AMP in juvenile NNH rats; Fig. 9c) and behavioral alterations indicating impulsivity (4.4±1.0, n = 7; t_6_ = 3.32, P = 0.023, unpaired t-test for SAL vs. AMP in NNH rats; Fig. 9d) and attention deficit (1.99±0.13 sec, n = 7; t_6_ = 2.79, P = 0.013 for SAL vs. AMP in NNH rats; Fig. 9e) in juvenile rats were also improved by AMP. Although NNH-induced behavioral alterations in juvenile rats were consistent with those caused by NHL, there were some differences between NNH- and NHL-induced behavioral alterations in adult rats. In particular, behavioral alteration indicating attention deficit in the object exploration test, which was present in adult NHL rats, was absent in adult NNH rat (1.99±0.20 sec, n = 6; Fig. 9e). In agreement with this observation, PL volume was smaller in juvenile NNH rats (0.90±0.01%, n = 5) than CTR rats (F_1,25_ = 18.5, P<0.001 for lesion types; F_1,25_ = 14.4, P<0.001 for ages; F_1,25_ = 5.09, P = 0.015 for lesion types×ages; Fig. 9f); however, different from NHL, PL volume reduction was not observed in adult NNH rats (0.94±0.02%, n = 5; Fig. 9f). Moreover, adult NNH rats exhibited attenuated locomotor response to AMP (Fig. 9c), which was opposite to adult NHL rats in which locomotor response to AMP was abnormally augmented (Fig. 2c).

These results suggest that neurodegeneration in the MHb, but not LHb, of neonatal brains is the primary root of the NHL-induced behavioral alterations observed during the juvenile period. In contrast, the neurodegeneraiton of the LHb in neonatal brains may be involved in behavioral and brain changes occurring in adulthood.

## Discussion

**Figure 10:**
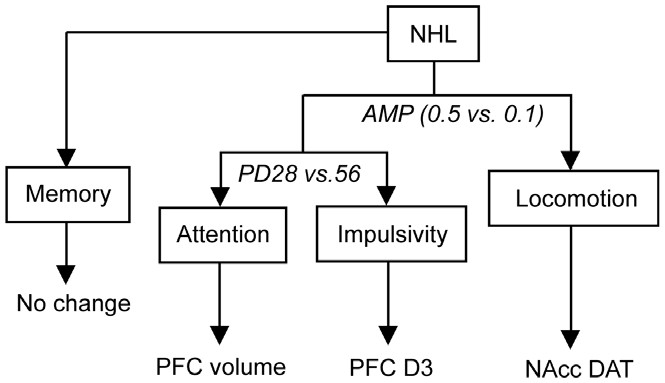
A schematic diagram illustrating behavioral and brain alterations caused by NHL.

In this study, we have shown that NHL induces neurodevelopmental sequelae of the cortico-striatal functions, which induces behavioral alterations resembling to the symptoms of ADHD. NHL induced hyper-locomotion and impulsivity in juvenile rats, which, however, disappeared when they reached adulthood. NHL also induced attention deficit, which was, on the other hand, present both in the juvenile period and adulthood. DA D3 receptor and DAT expressions were decreased in the PFC and NAcc, respectively, of juvenile, but not adult, NHL rats. PFC volume reduction was also observed in NHL rats, but in both the juvenile period and adulthood. We found that correlation existed between DAT expression in the NAcc and hyperlocomotion, whereas alterations within the PFC were correlated with impulsivity and attention deficit. Consistent with these observations, 0.5 mg/kg of AMP improved hyperlocomotion, but worsened impulsivity and attention deficit (Fig. 10). Attention deficit may be associated with PFC volume reduction that was present in both juvenile and adult NHL rats (Fig. 10), whereas impulsivity may be associated with a transient alteration in the PFC occurring only in the juvenile period such as D3 receptor expression (Fig. 10). Indeed, there were significant correlations selectively between DAT expression in the NAcc and locomotion, between PFC volume and performance in the attention test, and between D3 receptor expression and performance in the EPM. In the NAcc, DAT was decreased in the NAcc core in juvenile, but not adult, NHL rats. Such spontaneous hyper-locomotion and paradoxical attenuation of hyper-locomotion with AMP is consistent with those observed in DAT-knockout mice [47].

The habenula receives inputs from various brain areas including limbic structures, the basal ganglia, and the PFC, which in turn sends afferent projections to various brain areas such as the ventral tegmental area/substantia nigra and dorsal raphe, where DA and 5HT neurons are located, respectively. In this study, we specifically focused on NHL-induced alterations in the PFC and NAcc, given the suggested role of the extensive DA innovation into the PFC and NAcc on behavioral functions that we examined in this study such as locomotion, behavioral inhibition, and attention [48]. Studies have shown that the habenula provides inhibitory tone onto DA neuron activity and consequent DA release in the PFC and NAcc [1], [4], [5], [6], although its afferent projections to DA neurons are glutamatergic. This appears to be due to habenula projections selectively innervating onto GABA interneurons in the ventral tegmental area [2], [49] as well as indirect projections to DA neurons through the rostromedial tegmental nucleus [3]. As such, habenula lesion given in the adult brain induces DA-dependent behavioral manifestations including hyperlocomotion, impulsivity, and attention deficit [9], [10], [13], which are also induced by NHL. However, it is important to note that there is a major difference between adult habenula lesion and NHL. The characteristic feature of NHL-induced alterations is their presence in childhood, but disappearance in adulthood. Thus, disappearance of NHL-induced alterations at adulthood suggests that these alterations may not be caused simply by altered monoamine transmission, but by alterations specifically associated with neurodevelopment of the PFC and NAcc triggered by neonatal habenula insults. In particular, our study suggests that damage in the MHb is strongly associated with behavioral alterations observed during the juvenile period, as NNH, which induces neurodegeration in the MHb, but not LHb, causes behavioral alterations identical to those caused by NHL, which induces neurodegeneration both in the MHb and LHb. In contrast, there were some different alterations caused by NHL in adulthood, suggesting that the neurodevelopmental deficit in the LHb may be associated with emergence of alterations specifically during adulthood. Indeed, we also observed decrease of a number of DA receptor expressions in the NAcc, which, however, occurred only in adult NHL rats. Such post-pubertal DA receptor alterations in the NAcc may be associated with augmented locomotor responses to the higher dose of amphetamine that we observed in adult NHL rats.

D3 receptor expression in the PFC is of particular interest, as it is highly expressed in the PFC of neonatal brains, and decreases as animals grow. Such pattern of expression suggests that D3 receptor may play an important role on PFC function specifically in neonatal and juvenile periods. A similar pattern of D3 receptor expression has been also reported in the somatosensory cortex [50], [51], suggesting that such D3 receptor expression pattern is uniform across different cortical areas. Nevertheless, there is a study showing that it is D4, but not D3, receptor mRNA expression that is prominent in during the prenatal and neonatal periods and decreased through the postnatal life in the PFC of rodents [52]. The reason of discrepancy between this study and our finding is unclear.

Habenula deficits have been implicated in psychiatric disorders. Nevertheless, to date, no study has ever examined whether there is any abnormality in the habenula of ADHD brains. Indeed, no apparent brain damage has been observed in ADHD brains. However, it is interesting to note that antenatal maternal smoking during pregnancy has been shown to increase a risk of developing ADHD in off-spring [53], [54]. In particular, there are studies showing that repeated nicotine treatment induced neurodegeneraiton in the MHb [33]. Moreover, chronic nicotine treatment causes lesion of the fasciculus retroflexus, a bundle of output afferent fibers from the MHb, and decrease of cortical tone [55]. Therefore, although nicotine-induced MHb neurodegeneration reported in these studies is in adult, mature brains, it would be possible that maternal smoking during pregnancy may cause neurodegeneration selectively in the MHb of fetal brains, leading to neurodevelopmental disruption of the cortcio-striatal function and ADHD symptoms. Furthermore, increased prevalence of major depression has been shown in ADHD individuals, although such increased rate of depression in ADHD patients is suggested to be consequence of the social and interpersonal difficulties that they experience [56]. Nonetheless, an interesting coincidence is that there is strong evidence of habenula deficit in affective disorders [16], [17], [18], [19], [20], [21]. In summary, our study suggests that NHL would be a useful rodent model of ADHD. Future studies targeting the habenula in human subjects would be therefore a fruitful endeavor of research to understand pathogenesis and pathophysiology of ADHD.

## Methods

### Subjects and neonatal habenula lesion/neonatal nicotine microinfusion into the habenula

All experiments were conducted in accordance with the *Canadian Council on Animal Care Guide to the Care and Use of Experimental Animals* and were approved by the McGill University Animal Care and Use Committee (protocol # 5373).

Pregnant female Sprague-Dawley rats at gestational day 14 were purchased from Charles-River Laboratories, and housed individually in each cage. At PD7, female pups were culled, and NHL or NNH was performed in male pups. Pups were hypothermically anesthetized by placing them on ice for 10 to 15 minutes. Then, the pups were placed on a stereotaxic apparatus specifically designed for neonatal rats and mice, and incisions were made in the skull skin. Twenty six gauge Hamilton cannula connected to a microinfusion pump with silastic tube was lowered into the habenula (anterior/posterior: −1.8 mm posterior from bregma, medial/lateral: ±0.4 mm, dorsal/ventral: −3.2 mm below surface). This size of the cannula (O.D. = 0.46 mm, I.D. = 0.26 mm) was sufficiently small enough to induce lesion of the habenula without causing apparent damage in other brain structures. However, because of the size of the cannula, it was not possible to perform lesion restricted only in the MHb or LHb. Bilateral lesion of the habenula was given by infusion of 0.3 µl ibotenic acid (10 µg/µl dissolved in artificial cerebrospinal fluid [aCSF]) in each hemisphere at the speed of 0.15 µl/min. For microinfusion of nicotine into the habenula, 0.15 µg of nicotine dissolved in 0.3 µl aCSF was infused into each hemisphere of the habenula. After injection, cannula was kept in place for additional 3 minutes. CTR animals received sham lesion with infusion of the same volume of aCSF into the habenula. After injection, the incision was closed with clips, and the pups were recovered with a heat pad and returned to dams. We measured the areas of the MHb and LHb approximately at 3.3 mm posterior from the bregma to determine NHL. Because the size of the habenula significantly varied across anterior-posterior axis, we collected brain sections containing the habenula at the level where the most anterior tip of the ventral part of the third ventricles emerged immediately posterior to the central nucleus of the amygdala. We defined successful NHL as the areas of the LHb and MHb smaller than at least one standard deviation from the mean of CTR nuclei (Fig. 1c), whereas successful NNH was defined as the area of only the MHb smaller than at least one standard deviation from the mean of the nuclei in CTR rats. Neuronal densities of the MHb and LHb nuclei were examined in digitized photographs of Nissl stained sections using ImageJ software (particle analysis function; Fig. 3S).

### Locomotion and stereotypy tests

Horizontal locomotor activity was measured for 20 minutes in the open-field activity chamber (40×40×40 cm). Stereotypic behaviors were measured every 30 seconds for the first 10 minutes during the locomotion test. Stereotypy was quantified using the scaling system by Creese and Iversen [34].

### Effort- and delay-discounting tests

Impulsive behavior was further tested with effort- and delay-discounting tests using the T-maze that were modified from those used in the study by Denk [36].

In the effort-discounting test (Fig. 3a), rats made a choice of obtaining large (3 pieces of cereals) or small (1/2 piece of cereal) rewards located at the end of right and left arms. For testing in juvenile and adult rats, handling of animals was started on PD25 and PD53, respectively. Access to foods was also restricted to maintain the body weights to be ∼90% of age-matched animals with free food access. After 3 days of handling (15 minutes/day; in juvenile rats, this was stated from PD22) followed by habituation to the maze for another 3 days (15 minutes/day), animals were first trained to make a stable choice for large rewards over small rewards (i.e. >80% choice for large rewards in consecutive 10 trials) in the condition that animals freely accessed to both large and small rewards. On next day, the first session consisting of 10 trials was given to animals in which animals had again free access to both large and small rewards. Then, another session was given to animals subsequently after the first session in which a 5 cm barrier for juvenile rats (10 cm for adult rats) was now placed at the entrance of the arm for large rewards, such that animals had to across the barrier to reach to large rewards. This session was followed by two additional sessions in which the heights of barriers were further increased to 10 and 20 cm for juvenile rats (15 and 30 cm for adult rats) in the test.

In the delay-discounting test (Fig. 3c), the procedures for handling, habituation to the maze, and training were identical to those done in the effort-discounting test. After training, the first session was given in animals in which animals had free access to both large and small rewards. Then, another session was given to animals subsequently after the first session in which animals were held for 5 seconds in the middle of the arm by two clear acrylic guillotines between the entrance and the end of the arm where a large reward was located, whereas there was no delay period for reaching to a small reward. This session was followed by two additional sessions in which delays were prolonged to 15 and 25 seconds for large rewards. Percentages of choices for large rewards in each condition of the effort- and delay-discounting tests were measured.

### Elevated plus maze test

The EPM was used to examine impulsivity of animals. Animals were placed in the center of the elevated plus maze and their behaviors were monitored for 20 minutes.

The elevated plus maze (Fig. 3e) has been commonly used to test anxiety in rodents. The number of opened arm entries relative to enclosed arm entries is thought to be correlated with anxiety level, with increase/decrease of opened arm entries expressing less/more anxiety. Nonetheless, opened arm entries are also influenced by impulsivity [57] (Fig. 3j, Suppl. Fig. S1). Thus, when animals are unable to inhibit their inappropriate behaviors, they would exhibit increased opened arm entries regardless of anxiety level. Distinction of opened arm entries due to impulsivity or lower anxiety can be achieved by durations of time spent in the opened arms. If animals are less anxious, they would stay in the opened arm longer, whereas entering into the opened arm is due to impulsivity, these animals would return to the center more quickly (Fig. 3j, Suppl. Fig. S1).

### Color discrimination test

Attention deficit was tested with visual discrimination test modified from that used in the study by Petrof and Brown [37]. The color discrimination test was conducted in the open field chamber in which animals had to discriminate the two different colors, black and white, of bowls in which a reward was baited (Fig. 4a). After 3 days of handling followed by 3 days of habitation to the chamber, animals were first trained to discriminate the colors of bowls. Each piece of cereal was put in both black and white bowls hidden by chips to prevent use of odor cue. Animals were allowed to eat a cereal only in the black bowl, whereas when animals started to dig a white bowl, animals were immediately returned to a home cage without further digging. Bowls were located in the left and right corners of the chamber. Left and right positions of black and white bowls were pseudo-randomly changed in each trial to prevent use of spatial information. The training session was continued until animals made stable choice of digging a black bowl over a white bowl (i.e. >80% choice for large rewards in consecutive 10 trials). On next day, the first session consisting of 10 trials was given to animals in which animals had to again discriminate black and white bowls. Then, another session was given to animals subsequently after the first session in which a white bowl was now replaced to a gray bowl, such that animals had to increase attention level to discriminate the colors of bowls. Percentages of black bowl choices were measured in black-white and black-gray color bowl combination.

### Latent inhibition test

The conditioned attention theory proposed by Lubow [58] suggests that latent inhibition occurs as the subject is conditioned not to attend to the stimulus during pre-exposure, such that when subjects have attention deficit, they exhibit attenuated latent inhibition. Thus, we examined latent inhibition in adult NHL rats with the serial color/visual-cue discrimination tests (Fig. 4c), which is an analogous test to that used in the study showing attenuated latent inhibition in ADHD subjects [38]. Animals were first trained to the black-white color discrimination, and then subjected for training of the visual-cue discrimination test. Animals were randomly assigned into two groups, one pre-exposed to the visual-cue during which they performed the color discrimination test (PE group), and the other not exposed to the visual-cue in the color discrimination test (NPE group). Rats in the NPE group were first trained for the black-white color bowl discrimination test until their performance reached to the criterion as described above. On the next day, these animals were subjected for the visual-cue discrimination test in which two identical, gray color bowls were placed in the chamber now. A reward was buried in one of the bowls, which was marked by the visual-cue (i.e. flag) attached to the bowl. Thus, animals had to discriminate the bowls not by the colors of the bowls, but by the visual-cue attached to the bowl. For rats in the PE group, the behavioral testing procedure was identical to that for the NPE group, except additional placement of the visual-cue without a bowl during which they performed the black-white color discrimination test. Thus, animals were passively pre-exposed to the visual-cue for the rewarding bowl in the subsequent visual-cue discrimination test.

### Object exploration test

Sustained attention was examined with the task modified from object recognition test, which is similar to that used in the study by Lukaszewska [39]. In this test, 4 distinct features of objects were placed in the open field chamber (Fig. 4e), and animals were allowed to explore them for 10 minutes. Duration of time that rats spent on object exploration at each visit was measured. We reasoned that animals directed and sustained attention to these novel objects while they were exploring them. If animals are unable to sustain attention, duration of object exploration would be shorter. This was supported by the observation that administration of scopolamine (0.3 mg/kg, i.p.), the drug shown to impair attention [39], [59], decreased duration of time for object exploration in normal adult rats (Fig. 4h). Duration of time spent on object exploration may be also influenced by motivation of animals to explore them. To address this issue, the number of visits to objects was also counted.

### Novel object and place recognition tests

Non-spatial and spatial long-term memory tests were performed with object recognition test and its modified version, respectively [40] (Fig. 5a, b). In non-spatial test, animals explored two identical features of objects in the open field chamber for 10 minutes on first day. Twenty-four hours later, animals were placed in the open field chamber again for 10 minutes to explore objects, one that had been previously exposed on the first day and the other, new object. The ratio of number explored on the new object to pre-exposed object was measured. Spatial memory test was similar to the object recognition test. However, in this test, two objects used in the second exposure were identical to those used in the first exposure (thus, no new object), but placement of one of the two objects in the open field chamber in the second exposure was different from placements of objects in the first exposure. Spatial cues were provided on the wall of the chamber, such that animals realized the differences of object locations. It has been shown that animals explored more number on the object located in the new place [40].

### Drug treatments

d-amphetamine and apomorphine were dissolved in 0.5 ml of 0.9% saline. Drugs were given to animals (i.p.) 5 minutes before starting behavioral tests. CTR rats received 0.9% saline only.

### Quantitative PCR

Tissues from the PFC or NAcc were collected from NHL rats or sham CTR rats at PD9, 28 or 56, and the tissues were processed for quantitative PCR. RNA extraction was performed using Purezol RNA isolation regeant followed by Dnase I treatment using Aurum total RNA fatty and fibrous tissues kit. Then, cDNA conversion was performed using iScript cDNA synthesis kit. Primers were directed against the DA receptors, DAT, and a reference moclecule (β_2_-microglobulin) using the sequences shown in the Table 1. A mix of cDNA and 0.5 µM of the sense and antisense primers in LightCycler 480 SYBR Green 1 Master consisting of MgCl_2_, dNTPs, 2X SYBR Green I was loaded into Bio-Rad iCycler capillaries. The qPCR protocol consisted of initial HotStart Taq DNA polymerase activation cycle (5 minutes, 95°C, with a temperature transition rate of 20°C/sec) followed by 45 cycles of denaturation (10 seconds, 95°C), annealing (10 seconds, 61°C) and elongation (10 seconds, 72°C). A single fluorescence reading was acquired at the end of each elongation step. Subsequently, melting curve analysis were performed by heating to 95°C for 0 s, then immediately cooling to 60°C for 30 s, followed by a temperature increase to 95°C (temperature transition rate of 0.5°C/s), while continuously collecting the fluorescence signal data. The presence of a single melting peak followed by analysis on 1.5% agarose gel confirmed product specificity. Reactions were repeated in triplicate. Reactions were also carried out in the absence of reverse transcriptase to verify the absence of genomic DNA contamination. To determine the relative concentrations of mRNA gene expression, a standard curve of 5-fold serial dilutions of a mixture of each of the sample cDNA was used to plot the relative count value for each gene on the y-axis and the amount of cDNA used on the x-axis. To calculate the fold-change, the relative amount of mRNA product was divided by the relative amount of β_2_-microglobulin for DA receptors and DAT. Data analysis was conducted with the method developed by Pfaffl using REST 2008 software [60].

**Table 1:** Primer sequences used in qPCR.

Targets	Fwd sequences	Rev sequences	Efficiency
DRD1	CCT TCG ATG TGT TTG TGT GG	GGG CAG AGT CTG TAG CAT CC	1.026
DRD2	TCC CAG CAG AAG GAG AAG AA	GTG AAG GCG CTG TAG AGG AC	1.045
DRD3	CTA GTG GCC ACG TTG GTG AT	TGA CAT CCA GGG TGA CAA AA	0.889
DRD4	CTG CAG ACA CCC ACC AA CT	CCA TGA GGG TGT CAC AGA GG	1.051
DRD5	GCT GGG ATT ACA GAG GCA AC	ATG GCA GCA CAC ACT AGC AC	0.989
DAT	TGC TGG TCA TTG TTC TGC TC	ATC CAC ACA GAT GCC TCA CA	0.894
β_2_-microglobulin	CCG TGA TCT TTC TGG TGC TT	GGT GGG TGG AAC TGA GAC AC	0.904

### Western blot

Western blots were conducted to examine expressions of D3 receptor in the PL and DAT in the NAcc core. Animals were deeply anesthetized with a lethal dose of sodium pentoberbital (100 mg/kg), and transcardially perfused with ice-cold 0.1 M phosphate buffer saline (PBS). Brains were then removed from skulls, and tissues in the PL and NAcc core were isolated from other parts of the brains structures. Brain tissues were sonicated in RIPA buffer (Tris–HCl, 50 mM, pH 7.4; 1% Triton-X; 0.2% Na-deoxycholate; 1 mM EDTA; 0.2% sodium dodecyl sulfate; 1 µg/ml each of Aprotinin, leupeptin, and pepstatin), and centrifuged at 10,000×g. Then, protein levels in the supernatants were measured using Coomassie Plus Assay kit. Samples were normalized to be 16 (for NAcc core) or 18 (for PL) µg of proteins, run on a NuPAGE 4–12% Bis–Tris gel with a constant voltage (120 V), and electrophoretically transferred onto a nitrocellulose membrane using a semi dry blotter with a constant voltage (30 V) for 90 minutes. After 1 hour incubation in PBS with 5% non-fat milk to block non-specific binding, the membranes were incubated with primary antibodies targeting D3 receptor and DAT overnight at 4°C. Following several wash steps with PBS/Tween 20 (0.05%), membranes were incubated with horse radish peroxidase conjugated goat anti-rabbit or anti-mouse secondary antibody for 2 hours. Bands were detected by 5 min incubation with ECL plus. In order to exclude potential variation in protein estimation and gel loading, relative optical density were calculated for quantifications of D3 receptor and DAT expressions by comparison to expression of β-actin. ECL-treated membranes were scanned with the Typhoon Trio+ scanner, and digitized for quantitative analysis of receptors expressions. Optodensitometry quantification of the bands (intensity×area) was performed on these digitized data using ImageJ software.

### Histology

At PD28 or 56, animals were deeply anesthetized with a lethal dose of sodium pentoberbitol (100 mg/kg), and transcardially perfused with ice-cold 0.1 M PBS followed by 4% paraformaldehyde. Brains were removed from skulls, and post-fixed with paraformaldehyde. Then, brains were cryoprotected by 30% sucrose solution, and cut into 40 µm sections with microtome. These sections were processed for either free-floating SMI-32 or Nissl staining. SMI-32 immunohistochemistry was conducted to measure the volume of the anterior cingulate cortex (Cg1) as described in the study by Boire et al. [61] (Fig. 7a, b). Sections were washed in PBS and incubated with 0.5% H_2_O_2_ for 15 minutes to remove endogenous peroxides. Then, sections were incubated with 2.5% horse serum to block non-specific binding of antibody. SMI-32, the 200 kD primary antibody against a non-phosphorylated neurofilament epitope was incubated with sections for overnight at 4°C. Sections were further incubated secondary biotinylated anti-mouse IgG for 2 hours followed by ABC reagent for 2 hours (Vectastain Elite ABC kit, Vector Laboratories). Sections were then visualized by reaction with 3′3-diaminobenzidine. Sections were then mounted on slide glasses and hydrated by ethanol and xylene. Antibody binding to SMI-32 molecules was examined by a light microscope connected to CCD camera. Nissl-stained sections were used to measure the volumes of the medial PFC (prelimbic [PL] and infralimbic [IL] cortex) and orbitofrontal cortex (OFC; lateral [LO]+ventral [VO] orbitofrontal cortex; Fig. 7a, b). Anterior-posterior extent of the areas measured was from the level that the forceps minor of the corpus callosum started (approximately +4.6 mm anterior from the bregma, according to the atlas of Paxinos and Watson [62] to the level before the genu of the corpus callosum emerged (approximately +2.5 mm from the bregma). The prelimbic cortex was divided into the anterior and posterior parts at the point where the caudate emerged (approximately +3.0 mm from the bregma) for analysis of the volumes separately. Boundaries of PL and IL were identified with the various landmarks such as the azygous pericallosal artery (azp), cingulum (cg), fmi, and the genu of the corpus callosum, as other studies have examined the volumes of these brain areas with Nissl staining [63], [64]. Further methodological details for delineation of the Cg1, PL, and IL borders were described in Suppl. Fig. S4. The volume of the areas were computed according to V = (n−1)∑(i = 1) (A_i_+A_i+1_)/2×d_i_, where A_i_ is surface area in the i-th section, n is a number of sections, and d_i_ is distance between surface area A_i_ and A_i+1_ ( = 40 µm) [64]. A volume of a measured area was expressed as a percentage relative to a whole brain volume, V_w_. A total brain volume was determined by the Archimedes' principle while a whole brain was placed in sucrose solution for dehydration for histology, which was m = ρV_w_, where ρ was the mass density of sucrose solution (1.144 kg/m^3^ for sucrose solution used in our study) and m was weight of a whole brain.

### Data analysis

All data were expressed as means ± s.e.m. Unless otherwise specified, statistical significance was determined using multivariate ANOVA with repeated measures with lesion types (NHL or NNH vs. CTR sham lesion), ages of animals (PD28-35 vs. PD56 or older), and drug treatments (e.g. AMP treatments) as independent measures. Post-hoc analysis was conducted with Tukey test. Probability value of p<0.05 was considered to represent significant differences.

## Supporting Information

Figure S1 Graphs showing the effects of selective serotonin reuptake inhibitor (SSRI), fluoxetine, on the EPM test. It is generally considered that the EPM is the test to anxiety, such that animals are more likely to enter into the opened arms as well as spend more time in the opened arms if animals are more anxiolytic. We found that juvenile NHL rats exhibited increased entry into the opened arms but not the amount of time spent in the opened arms per visit. Such behavioral changes may not be explained only by change of anxiety. Thus, the number of opened arm entry may be associated with impulsivity rather than anxiety, whereas time spent on the opened arms may be associated with anxiety. Indeed, there has been an allegation that some aspects of behavior in the EPM test may be associated with impulsivity [57], but it has been rather ignored. To further address whether alterations observed in juvenile NHL rats was associated with impulsivity or anxiety, we examined the effects of SSRI, which has been shown to decrease impulsivity [65], but also produce anxiolytic effects [66], in normal juvenile rats. Thus, SSRI was expected to decrease the number of opened arm entry, but increase time spent in the opened arms per visit, if opened arm entry is associated with impulsivity, but time spent in the opened arms is associated with anxiety. (**a**) The SSRI, fluoxetine (10 mg/kg, ip; n = 6), decreased the number of entry into the opened arms in juvenile rats compared to those receiving saline (SAL; n = 6; unpaired t-test, t_10_ = 4.88, *P<0.001 for SAL vs. SSRI). Although decreased number of opened arm entries is thought to be reflecting increased anxiety, the effect of SSRI is suggested to be opposite, i.e. alleviation of anxiety. Thus, SSRI-induced decrease of opened arm entry cannot be explained by alteration of anxiety level, but by alteration of other behavioral component that influences opened arm entry, such as impulsivity. (**b**) Since the SSRI decreased the number of opened arm entry to almost no entry during the test, it was difficult to reliably assess the amount of time spent in the opened arm per entry in the SSRI-treated rats. Thus, immediately after the EPM test, we examined the amount of time spent in the opened arm in both SSRI- and SAL-treated rats by forcing them to go into the opened arm several times, and measured how long they spent in the opened arms until they returned to the center of the maze. SSRI significantly increased the amount of time spent in the opened arms per each entry (t_10_ = 2.70, **P = 0.022 for SAL vs. SSRI). In contrast, SSRI did not alter the number of entries and time spent per entry into the enclosed arms. These alterations suggest that opened arm entry is more strongly associated with impulsivity, whereas time spent in the opened arm is associated with anxiety.
(TIF)

Figure S2 Western blot assays for PFC D3 receptor and NAcc DAT expressions. (**a**) Top photographs show representative bands of D3 receptor and β-actin (β-act) in the PL. A bottom graph shows relative optical density (ROD) of D3 receptor expression over β-actin in the PL of juvenile NHL and CTR rats. *P = 0.046. (**b**) Top photographs show representative bands of DAT receptor and β-actin in the NAcc core. A bottom graph shows ROD of DAT expression over β-actin in the NAcc core of juvenile NHL and CTR rats. **P = 0.026.
(TIF)

Figure S3 Neuronal density of the LHb nuclei of adult NNH and CTR rats. A graph shows no difference of LHb neuronal density (a number of cells in 100 µm^2^ [10×10 µm]) between NNH and CTR rats.
(TIF)

Figure S4 Delineation of the borders of the Cg1, PL, and IL. (**a**) Two representative examples of the Cg1 with SMI-32 staining. SMI-32 resulted in fiber-like staining. The Cg1 area was determined by alignment of these fiber-like staining. One end of the boarder was defined by fiber-like staining (illustrated by the yellow lines) whose alignment was toward the tip of the forceps minor of the corpus callosum (fmi). The other end of the boarder was defined by the place where there was sudden change of alignment of fiber-like staining from curving oblique (illustrated by the red lines) to straight, horizontal (illustrated by the blue lines) orientation. (**b**) Three representative examples of the PL and IL along the anterior–posterior axis with Nissl staining. One end of the PL border (red arrows and lines) is at the point where sudden curve of the fmi starts. Since it can be seen with high magnification that Nissl stained cells are aligned straight from the fmi to the surface, one straight line that is originated from the point of the fmi with the red arrows can be determined (red lines) as the border. The other PL border is also one of the IL borders (blue lines). In the anterior part (∼3.7 mm anterior from the bregma), this border is the horizontal straight line starting from the ventral tip of the fmi to the surface of the cortex. In the more posterior part (∼3.2 mm), this border is defined with the hoziontal line starting from just dorsal to the azp hole (black arrow). In the most posterior part (∼2.8 mm), the border is at the slight change of the cg shape from oblique to more vertical line. The ventral border of the IL in the anterior part is determined the horizontal line started where the hole for the azp on the surface of the cortex (yellow line). In the more posterior part, the border starts at the ventral tip of the fmi. The ventral border of the IL at the most posterior part is defined at the point where there is very slight winding of the cg (which becomes more prominent in further posterior part).
(TIF)
