# Supplementary figures and images for: Neurodevelopmental Disruption of Cortico-Striatal Function Caused by Degeneration of Habenula Neurons
Source: PLoS One. 2011 Apr 29;6(4):e19450. doi: 10.1371/journal.pone.0019450 (PMC3084869; doi:10.1371/journal.pone.0019450)

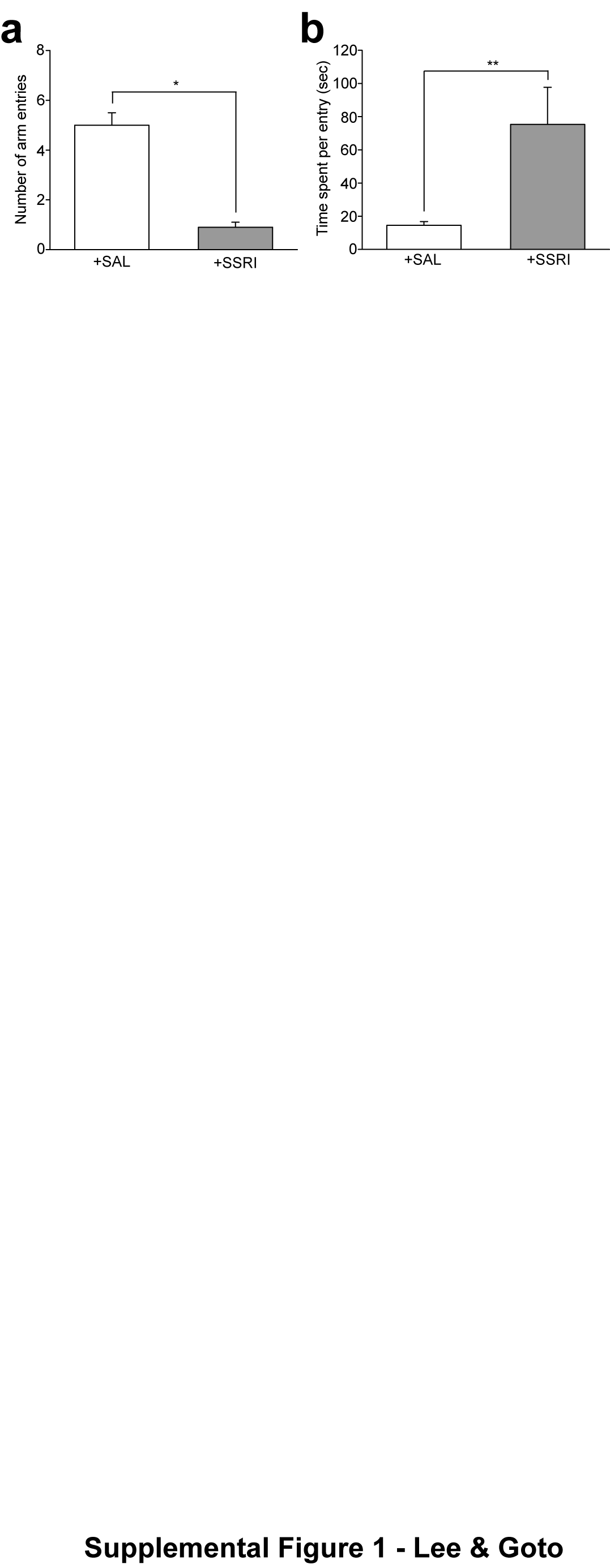

Supplement: Figure S1 — Graphs showing the effects of selective serotonin reuptake inhibitor (SSRI), fluoxetine, on the EPM test. It is generally considered that the EPM is the test to anxiety, such that animals are more likely to enter into the opened arms as well as spend more time in the opened arms if animals are more anxiolytic. We found that juvenile NHL rats exhibited increased entry into the opened arms but not the amount of time spent in the opened arms per visit. Such behavioral changes may not be explained only by change of anxiety. Thus, the number of opened arm entry may be associated with impulsivity rather than anxiety, whereas time spent on the opened arms may be associated with anxiety. Indeed, there has been an allegation that some aspects of behavior in the EPM test may be associated with impulsivity [57], but it has been rather ignored. To further address whether alterations observed in juvenile NHL rats was associated with impulsivity or anxiety, we examined the effects of SSRI, which has been shown to decrease impulsivity [65], but also produce anxiolytic effects [66], in normal juvenile rats. Thus, SSRI was expected to decrease the number of opened arm entry, but increase time spent in the opened arms per visit, if opened arm entry is associated with impulsivity, but time spent in the opened arms is associated with anxiety. (a) The SSRI, fluoxetine (10 mg/kg, ip; n = 6), decreased the number of entry into the opened arms in juvenile rats compared to those receiving saline (SAL; n = 6; unpaired t-test, t10 = 4.88, *P<0.001 for SAL vs. SSRI). Although decreased number of opened arm entries is thought to be reflecting increased anxiety, the effect of SSRI is suggested to be opposite, i.e. alleviation of anxiety. Thus, SSRI-induced decrease of opened arm entry cannot be explained by alteration of anxiety level, but by alteration of other behavioral component that influences opened arm entry, such as impulsivity. (b) Since the SSRI decreased the number of opened arm entry [file pone.0019450.s001.tif]

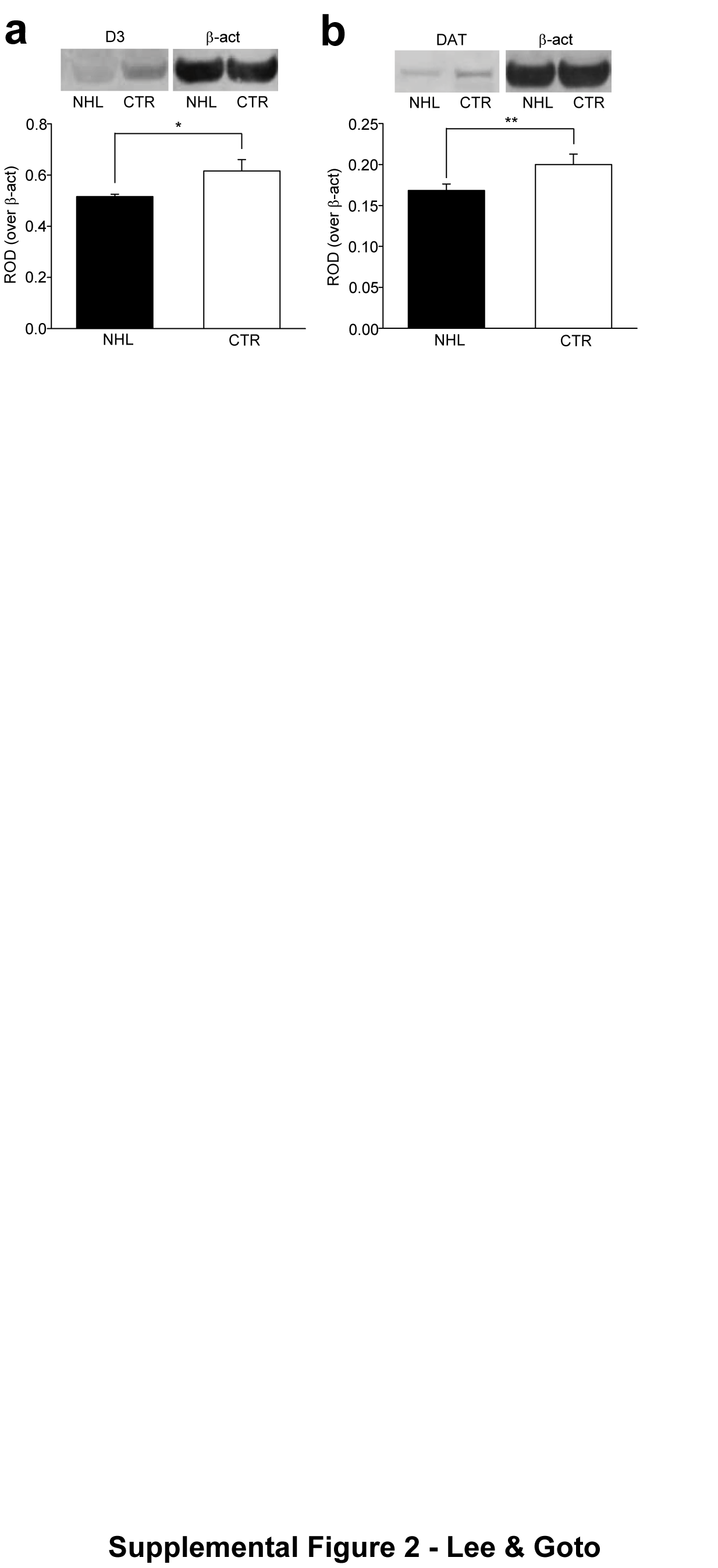

Supplement: Figure S2 — Western blot assays for PFC D3 receptor and NAcc DAT expressions. (a) Top photographs show representative bands of D3 receptor and β-actin (β-act) in the PL. A bottom graph shows relative optical density (ROD) of D3 receptor expression over β-actin in the PL of juvenile NHL and CTR rats. *P = 0.046. (b) Top photographs show representative bands of DAT receptor and β-actin in the NAcc core. A bottom graph shows ROD of DAT expression over β-actin in the NAcc core of juvenile NHL and CTR rats. **P = 0.026. (TIF) [file pone.0019450.s002.tif]

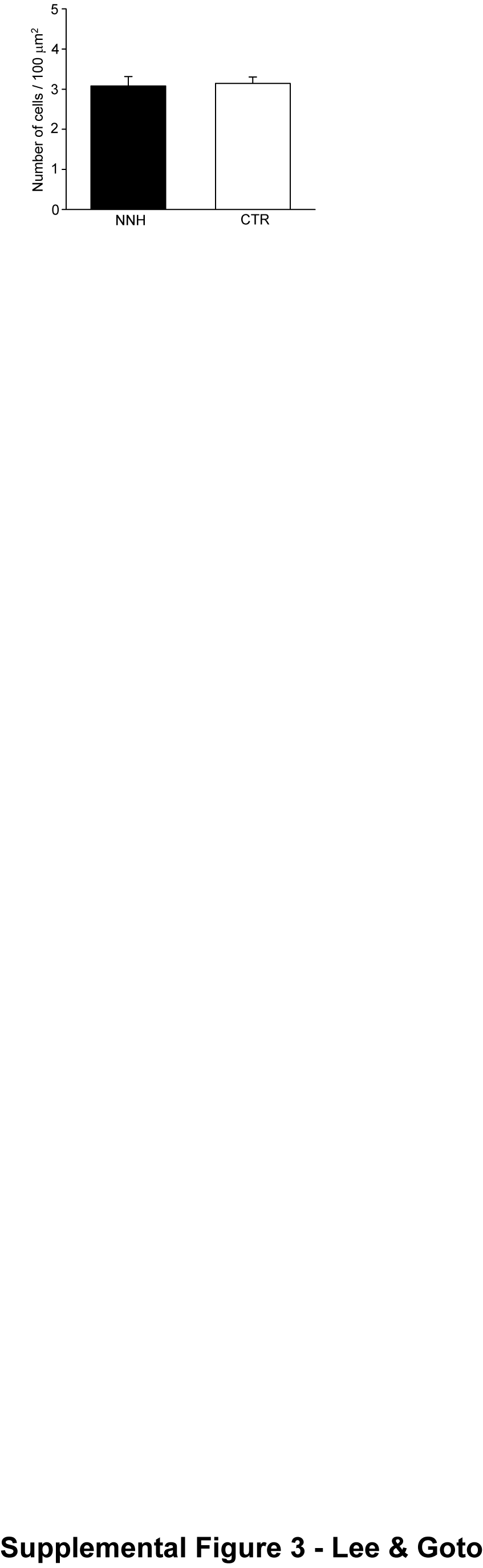

Supplement: Figure S3 — Neuronal density of the LHb nuclei of adult NNH and CTR rats. A graph shows no difference of LHb neuronal density (a number of cells in 100 µm2 [10×10 µm]) between NNH and CTR rats. (TIF) [file pone.0019450.s003.tif]

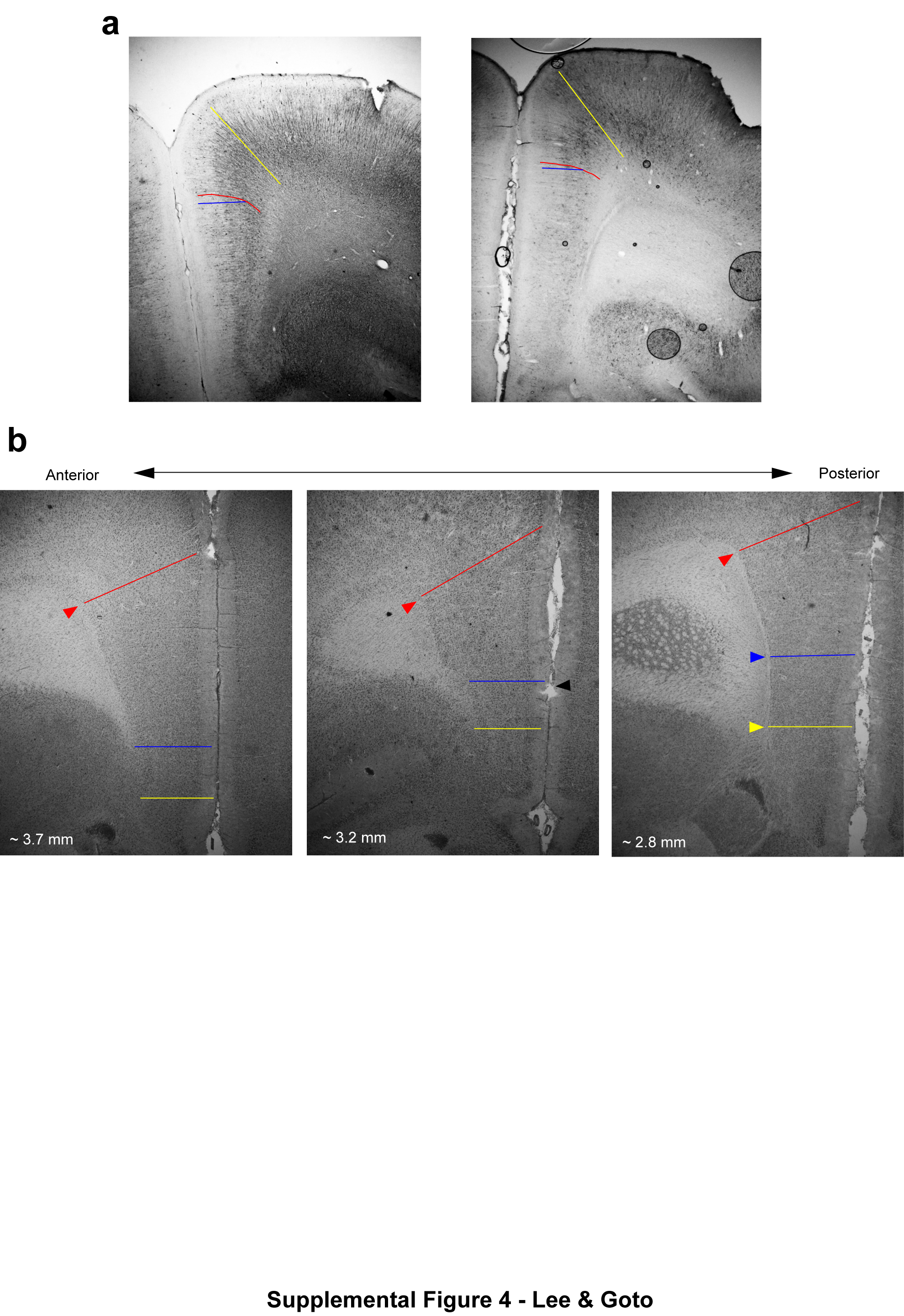

Supplement: Figure S4 — Delineation of the borders of the Cg1, PL, and IL. (a) Two representative examples of the Cg1 with SMI-32 staining. SMI-32 resulted in fiber-like staining. The Cg1 area was determined by alignment of these fiber-like staining. One end of the boarder was defined by fiber-like staining (illustrated by the yellow lines) whose alignment was toward the tip of the forceps minor of the corpus callosum (fmi). The other end of the boarder was defined by the place where there was sudden change of alignment of fiber-like staining from curving oblique (illustrated by the red lines) to straight, horizontal (illustrated by the blue lines) orientation. (b) Three representative examples of the PL and IL along the anterior–posterior axis with Nissl staining. One end of the PL border (red arrows and lines) is at the point where sudden curve of the fmi starts. Since it can be seen with high magnification that Nissl stained cells are aligned straight from the fmi to the surface, one straight line that is originated from the point of the fmi with the red arrows can be determined (red lines) as the border. The other PL border is also one of the IL borders (blue lines). In the anterior part (∼3.7 mm anterior from the bregma), this border is the horizontal straight line starting from the ventral tip of the fmi to the surface of the cortex. In the more posterior part (∼3.2 mm), this border is defined with the hoziontal line starting from just dorsal to the azp hole (black arrow). In the most posterior part (∼2.8 mm), the border is at the slight change of the cg shape from oblique to more vertical line. The ventral border of the IL in the anterior part is determined the horizontal line started where the hole for the azp on the surface of the cortex (yellow line). In the more posterior part, the border starts at the ventral tip of the fmi. The ventral border of the IL at the most posterior part is defined at the point where there is very slight winding of the cg (which becomes more prominent [file pone.0019450.s004.tif]
